# Self-supervised clustering of mass spectrometry imaging data using contrastive learning†

**DOI:** 10.1039/d1sc04077d

**Published:** 2021-11-26

**Authors:** Hang Hu, Jyothsna Padmakumar Bindu, Julia Laskin

**Affiliations:** Department of Chemistry, Purdue University West Lafayette IN 47907 USA jlaskin@purdue.edu; School of Engineering Technology, Purdue University West Lafayette IN 47907 USA

## Abstract

Mass spectrometry imaging (MSI) is widely used for the label-free molecular mapping of biological samples. The identification of co-localized molecules in MSI data is crucial to the understanding of biochemical pathways. One of key challenges in molecular colocalization is that complex MSI data are too large for manual annotation but too small for training deep neural networks. Herein, we introduce a self-supervised clustering approach based on contrastive learning, which shows an excellent performance in clustering of MSI data. We train a deep convolutional neural network (CNN) using MSI data from a single experiment without manual annotations to effectively learn high-level spatial features from ion images and classify them based on molecular colocalizations. We demonstrate that contrastive learning generates ion image representations that form well-resolved clusters. Subsequent self-labeling is used to fine-tune both the CNN encoder and linear classifier based on confidently classified ion images. This new approach enables autonomous and high-throughput identification of co-localized species in MSI data, which will dramatically expand the application of spatial lipidomics, metabolomics, and proteomics in biological research.

## Abstract 2

Contrastive learning is used to train a deep convolutional neural network to identify high-level features in mass spectrometry imaging data. These features enable self-supervised clustering of ion images without manual annotation.

## Graphical abstract

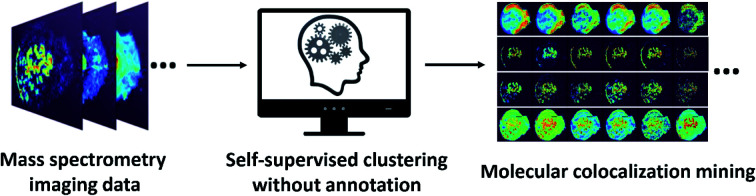

## Introduction

Mass spectrometry imaging (MSI) is a powerful label-free molecular imaging technique for biological research, which enables simultaneous localization of multiple classes of biomolecules with high sensitivity and unprecedented molecular specificity.^1–4^ By acquiring a full mass spectrum in each pixel of a virtual grid, MSI generates hundreds of molecular images in a single experiment. Recent advances in MSI technology focus on the enhancement of the spatial resolution,^5,6^ depth of molecular coverage^7–9^ and acquisition throughput,^10–12^ all of which substantially increase the data size. The interpretation of complex MSI data is a major bottleneck on the path to scientific discovery, which motivates the development of computational tools for data mining and visualization.^13,14^

A recurring task in MSI data analysis is to identify co-localized molecules, which is critical to the identification of key biochemical pathways of interest to biomarker discovery,^15,16^ drug development,^17,18^ and clinical diagnostics.^19–21^ Previous computational approaches used image vector-based similarity measurements to determine molecular colocalizations.^22–25^ However, these methods cannot correlate high-level spatial features making them disproportionately sensitive to the experimental artifacts and noise, which reduces their generalization capacity towards spatial patterns with similar localization but different contrast. Recently, transfer learning and semi-supervised deep learning approaches using convolutional neural network (CNN) have been developed to cluster ion images and quantify the molecular colocalization, respectively.^26,27^ These reports indicate that the limited size of MSI data presents a challenge to conventional CNN training frameworks, which typically rely on a large number of annotated images. As a result, these approaches provide a relatively minor improvement over the traditional machine learning methods for finding co-localized molecular images.

Recent advances in self-supervised contrastive learning approaches for computer vision including MoCo,^28^ SimCLR^29^ and SwAV^30^ have opened up new opportunities for learning visual representations without manual annotations. In natural image classification, these approaches provide comparable results to those obtained using supervised learning. In contrastive learning, image representations are learned by generating augmented instances of unlabeled images and using contrastive loss to minimize the difference between augmentations generated from the same image and maximize the difference between augmentations generated from different images. Following its success in computer vision, this strategy has been adopted in several applications in other research fields including classification of electrocardiograms^31^ and clustering of scRNA-seq data.^32^ It has been demonstrated that the development of modality-specific data augmentation is critical to the performance of models trained using contrastive learning.

Herein, we report on the development and performance of the contrastive learning approach for clustering of MSI data. We demonstrate that this strategy may be used to overcome the existing gap in the classification of MSI data due to the limited data size. We introduce a robust self-supervised clustering approach, which enables efficient colocalization of molecules in individual MSI datasets by retraining a CNN and learning representations of high-level molecular distribution features without annotations. The modality-specific data augmentation and classification fine-tuning methods were developed to build a fully unsupervised framework with optimal molecular colocalization performance.

## Results and discussion

### Self-supervised training of a CNN model for molecular localization clustering

**Fig. 1:**
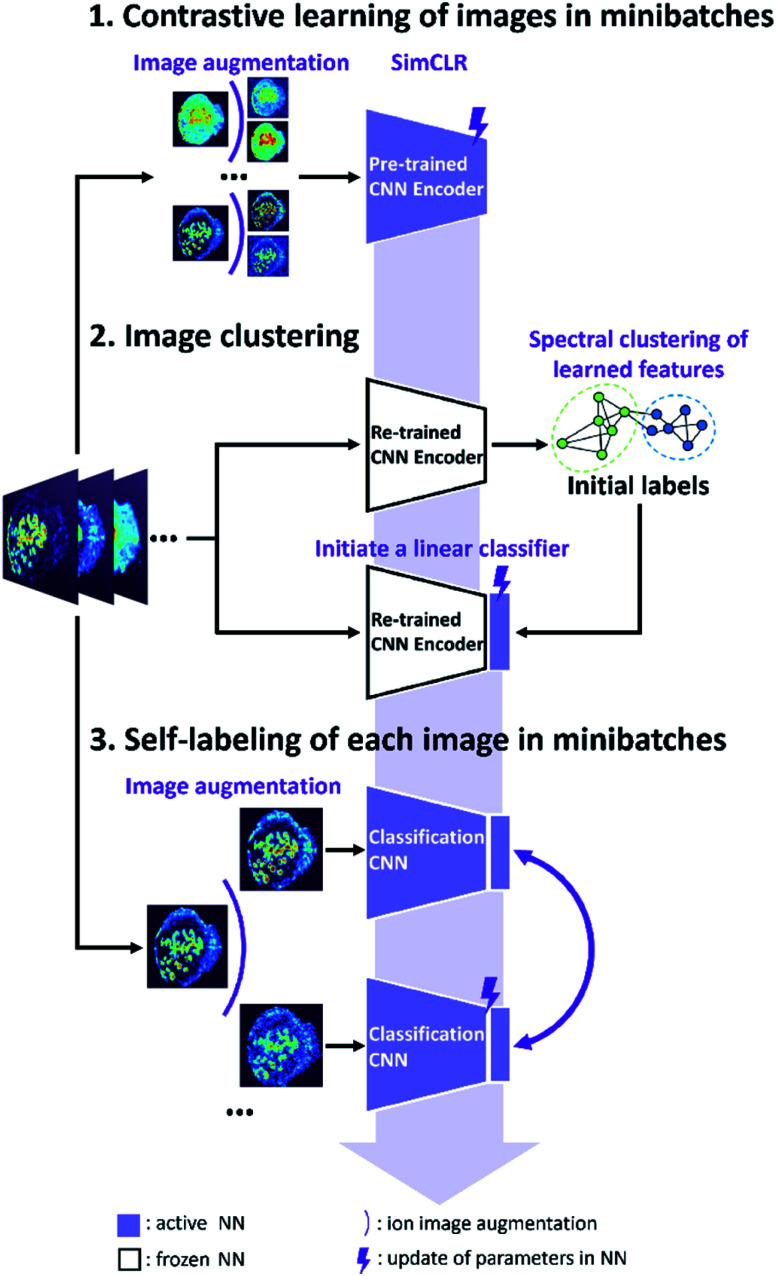
Self-supervised training of CNN model for molecular colocalization. (1) CNN encoder is trained by contrastive learning of images in minibatches to learn ion image representations. (2) Learned image representations are classified by spectral clustering. This classification pretext task is utilized to initiate a linear classifier after CNN encoder. (3) The classification CNN model is further fine-tuned by self-labeling of each image in minibatches. Black arrows indicate the data flow associated with images. The pale indigo arrow indicates the updating of CNN model across three steps of training.

The self-supervised approach for molecular colocalization developed in this study is illustrated in Fig. 1. The approach is based on training a CNN to learn representations of molecular localizations and classify molecular images into groups based on high-level spatial features. The clustering results provide a concise presentation of the spatial patterns present in large MSI data, which is critical to understanding the relevant biochemical pathways. To facilitate the autonomous and high-throughput MSI-based scientific discovery, we train our model in a self-supervised manner without manual annotations. This is achieved using image augmentation, which enables an effective self-supervised training of a deep CNN with a limited number of ion images. The self-supervised clustering approach developed in this study is summarized in Fig. 1. The approach relies on the following three steps described in detail later: (1) contrastive learning of molecular localization representations using SimCLR; (2) image clustering based on the learned representations and (3) self-labeling of the clustered images.

In order to assess the improvement of the model during the self-supervised training, we systematically evaluated each training step using a manually annotated benchmark MSI dataset of a mouse uterine tissue acquired using nanospray desorption electrospray ionization (nano-DESI).^33^ The mouse uterine tissue with several distinct cell types is an excellent model system, which presents diverse molecular localizations. From the data acquired in both positive and negative ionization modes, we manually selected 367 ion images (96 × 96 pixels) and clustered them into 13 classes (see ESI,† methods). We then validated our approach using a publicly available mouse brain tissue MSI dataset from METASPACE.^34^ It was acquired using matrix-assisted laser desorption/ionization (MALDI),^5^ which contains 1101 high resolution ion images (224 × 224 pixels) without annotations. Detailed dataset information are summarized in Table S1.† Our results demonstrate the robustness of the self-supervised clustering approach for MSI datasets of different sizes, spatial resolutions, tissue types, and acquisition conditions.

### Contrastive learning of image representations

In the contrastive learning step, we use SimCLR to train a CNN encoder for learning image representations. We used EfficientNet-B0 trained on ImageNet as a baseline CNN. EfficientNet^35^ has been demonstrated to achieve high accuracy on ImageNet and provide an order of magnitude higher efficiency than previous models, such as ResNet and Xception. In SimCLR framework (see ESI,† Fig. S1†), a mini-batch of N ion images is sampled and each image is subjected to a pair of stochastic transformations to generate 2N augmented images. A positive augmented pair is derived from the same ion image. Meanwhile, the remaining 2(N − 1) augmented images are treated as negative instances. SimCLR learns visual representations by maximizing the similarity between the positive pair of images while minimizing their similarity to the negative instances *via* a contrastive loss in the latent space. Details of the framework are described in the ESI† methods section.

**Fig. 2:**
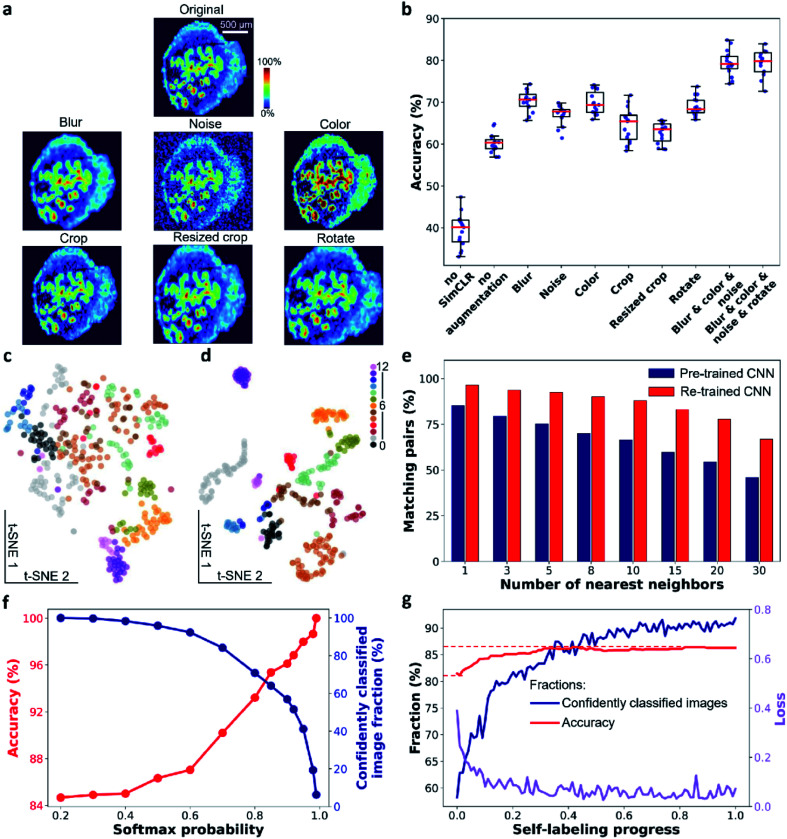
Self-supervised clustering enables effective molecular localization representation learning and classification in benchmark data. (a) Illustration of studied image augmentation operators. (b) Linear evaluation of the re-trained CNN encoder with individual or composite image augmentation operators. t-SNE visualizations of ion image representations obtained from (c) pre-trained CNN encoder and (d) re-trained CNN encoder. Each data point corresponds to an ion image. (e) Contrastive learning substantially improves the purity of local neighborhoods of ion images in the representation space. (f) The relationship between classification accuracy and fraction of confidently classified ion images, which are selected based on a series of softmax probability thresholds. (g) Changes in the training loss, accuracy, and number of confidently classified ion images during the self-labeling process.

Data augmentation plays a critical role in the training step. It ensures that the learned visual representations of ion images are independent of the employed transformations. This generalization power of SimCLR is critical to learning high-level spatial features instead of pixel-level details. In order to evaluate the performance of this step, we systematically investigated the impact of image augmentation operations on image classification in the benchmark dataset as shown in Fig. 2a. In particular, we used stochastic Gaussian blur, Gaussian noise, and intensity distortion to alter the appearance of ion images along with stochastic translation, resized crop, and rotation to alter their geometry. For each type of augmentation, we performed SimCLR using the same training protocol and evaluated the learned representations using a linear evaluation, in which the accuracy describes the quality of the representation (see ESI,† methods). We also examined the performance of a direct transfer learning (annotated as “no SimCLR” in Fig. 2b and S2b†) and SimCLR in the absence of augmentation (annotated as “no augmentation”) for comparison. Fig. 2b shows that all the appearance-changing augmentations improve the performance of the representation learning. Meanwhile, all the geometry-changing augmentations except for rotation do not provide a measurable improvement. Stronger geometric transformations reduce the classification accuracy (Fig. S2†). This observation indicates that in contrast to the semantic classification of natural images, strong alteration of the geometry of ion images is detrimental to representation learning of molecular localizations. We also examined the combined effect of the appearance-changing augmentations on the learned representations. Fig. 2b shows that a combination of three stochastic appearance-changing augmentation operators results in >80% accuracy in the linear evaluation. An example shown in Fig. S3† illustrates the power of the generalization provided by this augmentation strategy. In particular, for ion images that have different contrast and noise level, augmented images generated for one molecule (*m*/*z* 789.561 in positive mode) become similar to the original images of other molecules (*m*/*z* 746.5108 in positive and 599.3205 in negative modes, respectively). As a result, these molecules are classified into one group in the self-supervised clustering process. Our results indicate that, for MSI data, the generalization power of contrastive learning stems from the appearance-changing image augmentations.

The learned representations for the benchmark dataset are visualized using t-SNE in Fig. 2c and d with the color coding obtained from the manual image classification. The results demonstrate that the pre-trained EfficientNet-B0 model does not separate different classes of ion images (Fig. 2c). In contrast, both the separation and compactness of clusters are dramatically improved using the re-trained encoder (Fig. 2d). These findings indicate that contrastive learning provides meaningful localization representations, which may be used for image clustering without annotations. We also studied the impact of the training time on the learned representations as shown in Fig. S4.† Because the algorithm maximizes the similarity of positive pairs and minimizes the similarity of negative instances, we observe a trade-off between the alignment and uniformity in the learned image representations.^36^ Alignment indicates that feature vectors of two images from a positive pair should be mapped together while uniformity indicates that all feature vectors should be uniformly distributed. For the benchmark dataset, alignment dominates the training process in the first 50 epochs, in which ion images from the same class tightly aggregate together in the 2D feature space (Fig. S4a†). Further training beyond this point disproportionately increases the uniformity of the data distribution, which is detrimental to the downstream classification. In addition, a fast decrease in the contrastive loss observed in the first 50 epochs is followed by a much slower trend at longer training times (Fig. S4b†) indicating the diminished benefit of a longer training. The linear evaluation results shown in Fig. S4c† indicate that 50 epochs of training provide the best classification of the benchmark data.

### Image clustering

In the second step illustrated in Fig. 1, we performed image clustering based on the representations and generated the initial classification labels for the self-labeling task. Spectral clustering (SC) approach is selected, which constructs a *k*-nearest neighbor graph from ion image representations and then identifies clusters through the Laplacian embedding. Because contrastive learning provides image representations with meaningful local neighborhoods, SC is an appropriate method for this task.^37^ For representations of the benchmark dataset provided by contrastive learning, we quantified the purity of local neighborhoods by counting the annotation-matching pairs for each image and its *k*-nearest neighbors, where *k* ranges from 1 to 30 (see ESI,† methods). Our results confirm that contrastive learning substantially improves the purity of local neighborhoods of ion images in the representation space as shown in Fig. 2e. In particular, we observe that for a relatively large neighborhood size (*k* > 3), the re-trained encoder improves the pair-matching percentage by more than 15%. For example, for ten nearest neighbors, the pair-matching percentage is 88% and 66% for the re-trained and pre-trained encoders, respectively. In our implementation of the SC algorithm, we used ten nearest neighbors to construct the nearest-neighbor graph. The ten-neighbor condition provides a good balance between the connectivity and purity of each neighborhood, which are important to the data structure detection and clustering. As shown in Fig. 2e and Table S2,† the CNN encoder trained using SimCLR provides meaningful local neighborhoods for neighborhood sizes (*k*) ranging from 3 to 15. A combination of contrastive learning and SC provides 81.5% classification accuracy for benchmark dataset with 13 clusters as shown in Table 1. However, this machine learning classifier is non-learnable, which hinders further model improvement. In order to further enhance the clustering performance, we used the initial labels obtained from SC to initialize a learnable linear classifier at the end of the CNN encoder and then fine-tuned the model using a self-labeling approach.^38^ This classifier is composed of a linear layer followed by a softmax function. Its initialization is performed by training it on top of the frozen encoder with the original ion images and initial labels as inputs as illustrated in step 2 of Fig. 1.

**Table 1:** Summary of the performance of different clustering methods on benchmark data

Clustering setup	Number of clusters	Accuracy (%)
EfficientNet-B0 + SC	13	64.8 ± 0.4
SimCLR + SC	13	81.5 ± 3.4
SimCLR + SC + self-labeling	13	**84.0 ± 3.1**
EfficientNet-B0 + SC	20	71.9 ± 0.2
SimCLR + SC	20	90.0 ± 2.8
SimCLR + SC + self-labeling	20	**92.7 ± 2.1**

### Self-labeling

The self-labeling step shown in Fig. 1 fine-tunes both the CNN encoder and linear classifier by ensuring that augmentations of the same ion image are classified into the same group. This approach further enhances the generalization power of the model, which becomes tolerant towards visual variations originating from strong data augmentations (see ESI,† methods). To optimize the training process, only confidently classified images are included in self-labeling.

Because the initial labels are generated using an unsupervised machine learning approach, we anticipate that some false classification may be present. We identified falsely classified images based on their softmax probabilities.^39^ By excluding these images from training, we improved the robustness of the CNN model, which benefits the classification accuracy. In order to select images with correct classification during the training, we first examined the relationship between the softmax probability and classification accuracy for the CNN model using the benchmark dataset. This model was trained by initial labels obtained from SC and classified ion images into 13 classes. We used a range of softmax probability thresholds to examine the classification accuracy (red trace) and fraction of confidently identified images (blue trace) as shown in Fig. 2f. We observe that the classification accuracy increases with increase in the softmax probability threshold. Meanwhile, the number of confidently classified images decreases. Additional examples of this analysis are shown in Fig. S5† indicating that the observed trend is general.

The results shown in Fig. 2f indicate that there is a trade-off between the number of confidently classified images and classification accuracy. In self-labeling, we chose a probability threshold of 0.9 to start training, for which 58% of confidently classified images were selected with 96% classification accuracy. Self-labeling is performed by re-training both the CNN encoder and classifier using selected images. For each ion image, we use one weak and one strong data augmentation (see ESI, Table S3† and methods), which provides two pseudo labels as the classifier outputs. A cross-entropy loss is calculated for the pseudo labels and the model parameters are updated to minimize the loss as illustrated in Fig. 1. In each epoch, we update the confidently classified images for training using the same softmax probability threshold of 0.9. As illustrated in Fig. 2g, the loss (purple line) decreases with training time. Meanwhile we observe a significant increase in the number of confidently classified images and a slight increase in the accuracy with training time. These results demonstrate that the CNN model corrects itself during the self-labeling process, which gradually includes additional confidently classified ion images into the training and increases the overall classification accuracy.

**Fig. 3:**
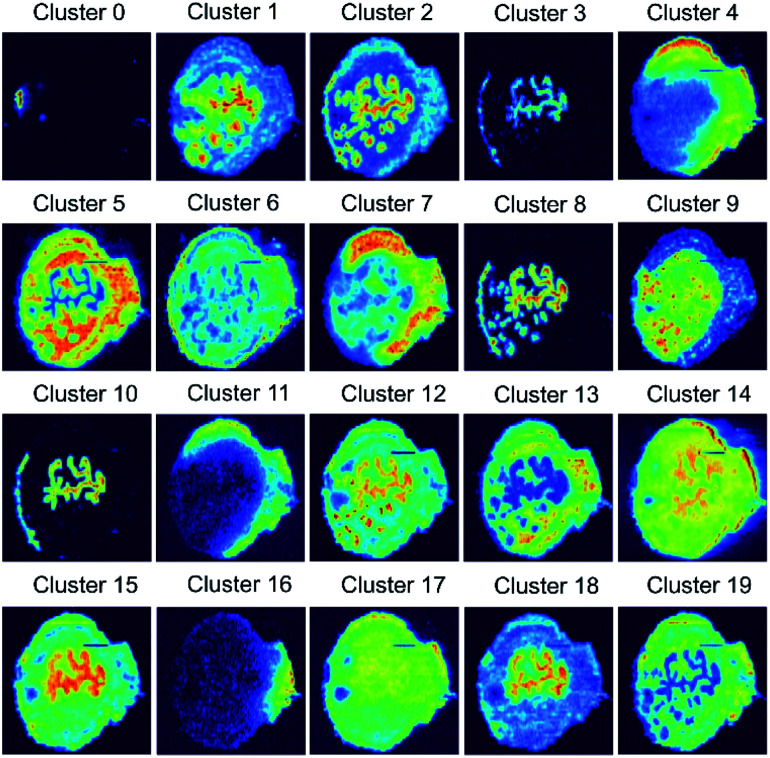
20 average ion images obtained from self-supervised clustering results provide a concise summary for comprehensive molecular distribution patterns present in the benchmark MSI data.

We used the self-supervised clustering approach to cluster benchmark ion images of the mouse uterine tissue (Fig. S6†) into 13 and 20 groups. The results obtained at different stages of the workflow for five replicates are summarized in Table 1. When clustering is performed using the CNN encoder and SC, contrastive learning (SimCLR) improves the classification accuracy from 64.8% to 81.5% with 13 clusters and from 71.9% to 90.0% with 20 clusters. An improvement of about 20% in accuracy clearly indicates the significance of the CNN retraining for learning image representations in MSI data. In addition, self-labeling provides a 3% improvement in the classification accuracy for both 13 and 20 clusters. Collectively, our self-supervised clustering approach enabled clustering of the benchmark data into 20 groups with 92.7% accuracy as shown in Fig. S7.† Representative ion images for each group shown in Fig. 3 provide a concise summary of the spatial patterns present in the vast MSI data. Meanwhile, the generalization power of the self-supervised clustering approach and its tolerance to noise levels can be assessed by examining images in each class of Fig. S7.†

### Comparison of the self-supervised clustering with vector-based methods

**Fig. 4:**
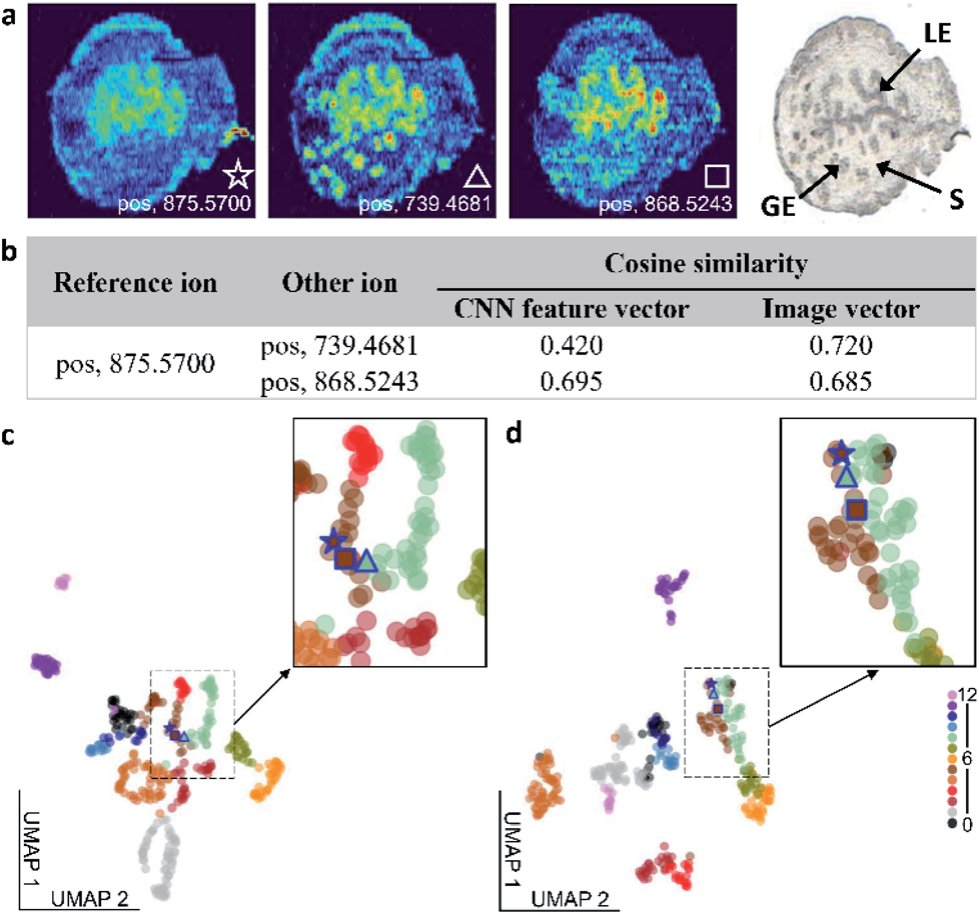
Comparison of the molecular colocalization measurements obtained using the self-supervised clustering approach and image vector-based methods. (a) Ion images of *m*/*z* 875.5700, 739.4681, and 868.5243 denoted with a star, triangle, and square, respectively, and an optical image from the benchmark dataset. (b) Cosine similarity scores for both CNN feature vectors and image vectors obtained for the three ion images. UMAP visualizations of (c) CNN feature vectors and (d) image vectors. Zoom-in regions show the location of the three ion images in panel a on the UMAP plot.

We compared the performance of the self-supervised clustering developed in this study with conventional vector-based approaches used in MSI. Although all the approaches used in this comparison rely on the similarity measurement between vectors for image classification, the classification accuracy shown in Table S4† varies between the methods. In the self-supervised clustering approach, the CNN encoder converts the high-level spatial information of the observed molecular distributions into feature vectors. These feature vectors are subsequently classified into distinct spatial patterns using either a clustering algorithm or an iteratively trained classifier. In contrast, vector-based clustering methods convert ion images into image vectors, which are subsequently subjected to clustering analysis.^24,25,40^ This flattening of the MSI data results in a substantial loss of the spatial information content, which makes vector-based methods disproportionately sensitive to the experimental artifacts and noise. To compare the performance of the self-supervised clustering approach with vector-based methods, we used ion image similarity measurements as illustrated in Fig. 4 and S8.†

In the example shown in Fig. 4a, we use an ion image of *m*/*z* 875.5700 as a reference and correlate it to images of *m*/*z* 739.4681 and *m*/*z* 868.5243. The pairwise cosine similarity scores obtained using both the CNN feature vectors, generated by the encoder shown in Fig. 2d, and image vectors are listed in Fig. 4b. Although ion images of *m*/*z* 875.5700 and 868.5243 indicate that these ions are enhanced in luminal epithelial (LE) cells, the signal of *m*/*z* 875.5700 in the LE region is relatively low and the distribution is less distinct than that of *m*/*z* 868.5243. In contrast, *m*/*z* 739.4681 is enhanced in both the LE and glandular epithelial (GE) cells. Therefore, we expect to obtain a better correlation between ion images of *m*/*z* 875.5700 and 868.5243 than between *m*/*z* 739.4681 and other two species. The pairwise cosine similarity scores obtained using the CNN feature vectors suggest that the reference ion has a better colocalization with *m*/*z* 868.5243 (0.695) than with *m*/*z* 739.4681 (0.420), which is consistent with the expectation. However, the similarity scores calculated using image vectors are clearly affected by the low intensities of the reference ion in the GE region and predict the opposite trend. This comparison confirms that the self-supervised clustering approach is substantially more tolerant to chemical noise than vector-based approaches.

We also used the Uniform Manifold Approximation and Projection (UMAP) algorithm to project both the CNN feature vectors and image vectors onto a 2D space, as shown in Fig. 4c and d. In these plots, each ion image is represented by a filled circle and color coded by their manual image classification. The three ion images shown in Fig. 4a are highlighted in the UMAP plot. In the UMAP plot obtained for the CNN feature vectors shown in Fig. 4c, *m*/*z* 875.5700 is mapped closer to *m*/*z* 868.5243 than to *m*/*z* 739.4681, which is in agreement with our expectations. In contrast, *m*/*z* 739.4681 is mapped between other two ions in the UMAP plot of image vectors shown in Fig. 4d. We also observe mixing between ion images from class 5 and class 8 in Fig. 4d. This further highlights challenges associated with image clustering using vector-based approaches, which may lead to errors in data structure visualized using UMAP analysis. A similar phenomenon is observed when the Ward hierarchical clustering is applied to image vectors shown in Fig. S9.† This analysis indicates that the Euclidean distance measurement cannot differentiate between the ion image of *m*/*z* 739.4681 and two other ion images used in this example. The biases in the similarity measurement using image vectors are observed for a range of ions, as shown in Fig. S8 and Table S5.†

In summary, the CNN feature vectors generated in SimCLR training provide a more accurate pairwise ion image similarity detection than vector-based methods. This is largely due to the strong generalization capability of the re-trained CNN, which identifies high-level spatial features even in noisy MSI data.

### Mass spectrometry image clustering of an unannotated mouse brain dataset

To further demonstrate the robustness of the self-supervised clustering approach, we applied it to a publicly available mouse brain MALDI MSI dataset. The image size of 224 × 224 pixels is larger than the benchmark data. For the mouse brain MSI data, we generated 1101 ion images shown in Fig. S10.† We observe diverse spatial patterns of metabolites and lipids localized to different regions of the brain tissue. Ion images showing signal enhancement outside of the tissue region most likely correspond to matrix peaks. Using self-supervised clustering approach, we re-trained the CNN model and clustered 1101 ion images into 35 colocalization groups as shown in Fig. S11.† This process took less than one hour with a single GPU card (see ESI,† methods).

**Fig. 5:**
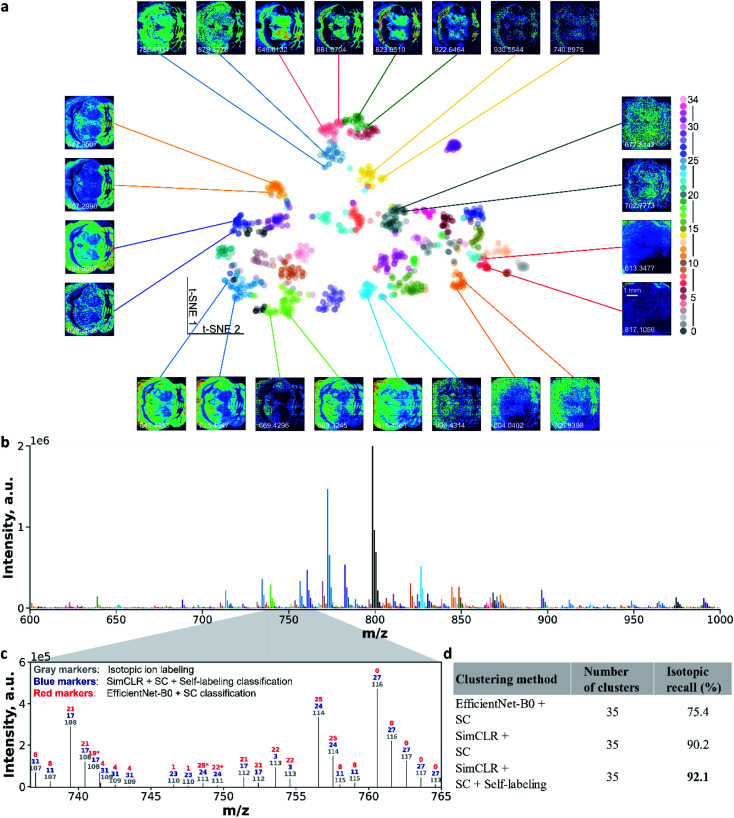
Self-supervised clustering results on a publicly available MALDI mouse brain dataset. (a) t-SNE visualization of ion image representations obtained after two steps of self-supervised training. Data points are color-coded using the final clustering assignments of ion images. Pairs of representative ion images are selected from well-resolved clusters to visualize the quality of classification. (b) An average spectrum color-coded using the same color scheme as that in panel a. (c) A zoom in region of the average spectrum showing several isotopic patterns. The results of isotopic identification (ground truth), EfficientNet-B0 and SC classification, and self-supervised clustering classification are annotated using independently assigned class numbers with different colors. Ions with the same color and number are grouped together in the corresponding classification. For clustering results, an asterisk indicates falsely classified isotopic ions. (d) A summary of the isotopic recall for different clustering methods.

Fig. 5a illustrates ion image representations after self-supervised learning using t-SNE visualization. Additional results are provided in Fig. S12.† In the absence of a manual annotation, we use the black color for all the data points in Fig. S12.† With the pre-trained EfficientNet-B0, we could only observe several aggregates at the edge of the 2D ion image representations. However, the uniformly distributed representations in the center of the plot cannot be used for identifying the co-localized ion images (Fig. S12a†). After the contrastive learning step, the re-trained CNN encoder provides a substantially improved separation of the representations as shown in Fig. S12b.†Fig. 5a shows ion image representations after self-labeling, which are color-coded with predicted colocalization labels. Tight clusters indicate co-localized molecular distribution patterns in the MSI data. Pairs of ion images were selected from clusters and placed around the t-SNE plot. Images from one pair have similar spatial features, while different pairs show distinct molecular localizations. These results confirm that the self-supervised clustering approach developed in this study provides accurate molecular localization representations of distinct spatial patterns observed in MSI data. Notably, some of the paired ion images have different noise levels (*e.g.*, *m*/*z* 906.4314 *vs. m*/*z* 915.4561) or different intensity levels (*e.g.*, *m*/*z* 613.3477 *vs. m*/*z* 817.1050). These results indicate that data augmentations used in the training step provide a sufficient generalization capability for the re-trained CNN model to identify high-level molecular localization. The ability to perform self-supervised clustering of the unannotated MSI data distinguishes our approach from previously reported methodologies.^24–27^

We also visualized the ion clustering results in the *m*/*z* space. Fig. 5b shows an average mass spectrum over the *m*/*z* 600–1000 range, in which peaks are highlighted using the same color coding as that in Fig. 5a. To further evaluate the accuracy of the clustering, we examined the isotopic recall,^26^ which quantifies the percentage of ion images of isotopic peaks correctly grouped together. We identified isotopic ions based on both the accurate *m*/*z* shift and Pearson correlations of ion images (see ESI,† methods). For example, in Fig. 5c, co-localized isotopic peaks observed in the *m*/*z* range of 736–765 are annotated using compound indices in gray color, which are ranked by their primary isotopic *m*/*z* values. Ion image colocalization results of self-supervised clustering and EfficientNet-B0 followed by SC are also annotated by colocalization class number with blue and red colors, respectively. We note that independent class numbers were assigned to these two classification results. With the expectation that isotopic images should be clustered into the same group, we identified the correctly and falsely classified isotopic ions in clustering results (see ESI,† methods) and marked false isotopic classification with an asterisk. In the mass range shown in Fig. 5c, EfficientNet-B0 and SC falsely classified 3 isotopic peaks, while the self-supervised clustering approach correctly grouped all the isotopic peaks. This result further confirms the accuracy and robustness of the self-supervised clustering. Values of the isotopic recall obtained using different clustering methods are summarized in Fig. 5d. For the clustering involving the CNN encoder and SC, contrastive learning (SimCLR) improves the isotopic recall from 75.4% to 90.2%. With the self-labeling, the final model reaches an isotopic recall of 92.1%, which indicates the superior clustering performance of this approach.

## Conclusion

We developed a robust self-supervised clustering approach for classifying co-localized molecular images obtained using MSI. In this approach, data augmentation is combined with contrastive learning and self-labeling methods to train a deep CNN model without manual annotations. Systematic studies using a fully annotated mouse uterine tissue data and unannotated mouse brain tissue data demonstrate that the re-trained CNN model efficiently learns high-level molecular localization representations, which facilitate clustering of molecular images. Using a manually annotated benchmark dataset, we demonstrate that this approach achieves >90% classification accuracy. Meanwhile, clustering of a publicly available unannotated MSI data demonstrates the robustness of this approach and its applicability to different tissue types, image sizes, modes of ionization, instrument parameters, and data complexity.

Our findings indicate that the limited size of MSI data is not a bottleneck for self-supervised learning. However, data augmentation is critical to the model training. We use a combination of stochastic appearance-changing transformations, such as Gaussian blur, Gaussian noise and intensity distortion to maximize the generalization power of the CNN model towards the efficient recognition of distinct localization patterns in ion images with varying levels of signal and noise. A similar self-supervised learning paradigm may be applied to other hyperspectral chemical imaging modalities including Raman and infrared microscopy.

The approach presented herein enables molecular colocalization analysis based on the MSI data in an autonomous and high-throughput manner. It provides a concise representation of the vast data containing several hundreds of molecular images, which is critical to understanding biochemical pathways in biological systems. Furthermore, we propose that this approach may be readily expanded into a larger semi-supervised learning framework. The self-supervised paradigm enables representation learning before supervised classification, which is particularly advantageous for automatic ion image labeling necessary for the high-throughput annotation of both MSI data and data obtained using other imaging modalities.

## Data availability

The mouse brain MSI dataset can be obtained from METASPACE (https://metaspace2020.eu). The dataset title is: Mousebrain_MG08_2017_GruppeF. Mouse uterine MSI benchmark data and the source code for the model training and inference are available on GitHub (https://github.com/LabLaskin/MSI-self-supervised-clustering).

## Author contributions

H. H. and J. L. conceptualized the project and methodology. H. H. and J. P. B. analysed the data and built data pipeline. H. H. collected experimental data and wrote the original draft. J. L. supervised the investigation and co-wrote the manuscript.

## Conflicts of interest

There are no conflicts to declare.

## Supplementary Material

SC-013-D1SC04077D-s001
