# Supplementary material for: Self-supervised clustering of mass spectrometry imaging data using contrastive learning
Source: Chem Sci. 2021 Nov 26;13(1):90–8. doi: 10.1039/d1sc04077d (PMC8694357; doi:10.1039/d1sc04077d)
Supplement: SC-013-D1SC04077D-s001 [file SC-013-D1SC04077D-s001.pdf]

Electronic Supplementary Material (ESI) for Chemical Science.
This journal is © The Royal Society of Chemistry 2021

Electronic Supplementary Information

Self-supervised Clustering of Mass Spectrometry Images Using Contrastive learning

Hang Hu[1], Jyothsna Padmakumar Bindu[2] and Julia Laskin[1*]

1. Department of Chemistry, Purdue University, West Lafayette, IN 47907, USA

2. School of Engineering Technology, Purdue University, West Lafayette, IN 47907, USA

Corresponding author: Julia Laskin, Tel: 765-494-5464, Email: jlaskin@purdue.edu

Table of content

1. Methods and data analysis………………………………………………….. S2 – S5

2. Additional tables

Table S1………………………………………………………………….....S6
Table S2………………………………………………………………….....S6
Table S3………………………………………………………………….....S6
Table S4………………………………………………………………….....S6
Table S5………………………………………………………………….....S7

3. Additional figures

Figure S1…………………………………………………………………...S8
Figure S2…………………………………………………………………...S8
Figure S3…………………………………………………………………...S9
Figure S4…………………………………………………………………...S9
Figure S5…………………………………………………………………...S10
Figure S6…………………………………………………………………...S11 – S12
Figure S7…………………………………………………………………...S13 – S14
Figure S8…………………………………………………………………...S15
Figure S9…………………………………………………………………...S15
Figure S10…………………………………………………………………S16 – S20
Figure S11…………………………………………………………………S21 – S26
Figure S12…………………………………………………………………S27

4. References…………………………………………………………………S27

Methods and data analysis

Mass spectrometry imaging data

Mouse uterine and brain tissue MSI datasets used as examples in this study have been previously reported.[1,2] Uterine tissues from 2-month-old female mice were snap-frozen, and frozen sections were made with a cryostat. The mice were maintained on a C57BL6 mixed background and housed in the vivarium at the Cincinnati Children's Hospital Medical Center according to NIH and institutional guidelines for laboratory animals. This protocol was approved by the Cincinnati Children's Hospital Research Foundation Institutional Animal Care and Use Committee. Mouse uterine tissue was analyzed using nano-DESI MSI on a Q-Exactive HF-X Orbitrap mass spectrometer (Thermo Fisher Scientific, Waltham, MA) equipped with a custom-designed nano-DESI source.[3] Mass spectra were acquired in the *m/z* range of 133-2000 in both positive and negative ion modes with a spatial resolution of 10 μm. Positive mode MSI data for mouse brain tissue was obtained from METASPACE,[4] a community resource that provides open access to MSI data. The specific data were acquired using MALDI MSI in the *m/z* range of 600-1000 with a spatial resolution of 20 μm. The dimensions of the two MSI datasets are listed in Table S1.

Data pre-processing

To generate ion images from MSI data, we used peak detection and *m/z* binning as described in our previous study.[5] The signal intensity for each *m/z* in each pixel was extracted from the corresponding mass spectrum with a bin width of ± 10 ppm and normalized to the total ion current. To remove visual spikes, pixels with intensities > 0.999 quantile were reassigned with the 0.999 intensity quantile value. Ion images of mouse uterine tissue were resized to 96 x 96 pixels. Meanwhile, larger-size ion images of mouse brain tissue were resized to 224 x 224 pixels. Most pre-trained CNN models and Pytorch transform functions accept RGB images. Thus, raw pixel intensities of ion images were normalized between 0 and 255 and copied to 3 channels. More specifically, we converted ion images into the PIL format before the data augmentation step. To benchmark our approach, we manually selected 367 mouse uterine ion images with distinct ion distributions and clustered them into 13 groups according to molecular colocalizations as shown in Fig. S6. For the unannotated mouse brain dataset, we detected 1101 peaks from the average spectrum and generated the corresponding ion images as shown in Fig. S8.

Architecture of the self-supervised clustering

We approached the challenge of molecular localization clustering as an image classification task. We aimed to re-train a CNN model for an individual MSI dataset to classify ion images based on the high-level spatial features without manual annotations. The model architecture is shown in Fig. 1. The pre-trained CNN (EfficientNet-B0) is re-trained by contrastive learning and self-labeling sequentially in a self-supervised manner. We use EfficientNet-B0, which has been trained on the ImageNet database. We used the EfficientNet-B0 model before the classification layer as an encoder. In our architecture, we firstly learned ion image representations through the contrastive learning. More specifically, SimCLR[6] approach is adopted in this study. After this first phase of training, we fed ion images through the re-trained encoder to produce a set of feature vectors, which were then passed to a spectral clustering (SC) classifier to generate the initial labels for the classification task. To complete a learnable classification CNN, a linear classifier (a linear layer followed by a softmax function) was attached to the encoder and trained with the original ion images and initial labels as inputs. Finally, we utilized a self-labeling[7] approach to fine-tune both the encoder and classifier, which allows the network to correct itself.

Contrastive learning

Details of SimCLR implementation are shown in Fig. S1. The representation of an ion image is the output before the classification layer of EfficientNet-B0. A small multilayer perceptron with one hidden layer maps the representations to the projection space (Z) where the contrastive loss is applied. In the training step each ion image is used to generate a pair of augmentations. We treat two augmentations of one ion image as a positive pair (i, j) . Then the loss function is defined as

$$\ell_{i,j} = -\log \frac{\exp\left(\mathrm{sim}\left(\mathbf{z}_i, \mathbf{z}_j\right)/\tau\right)}{\sum_{k=1}^{2N} \mathbf{1}_{[k \neq i]} \exp\left(\mathrm{sim}\left(\mathbf{z}_i, \mathbf{z}_k\right)/\tau\right)}$$

where 2N is the number of augmented images, $\mathbf{z}_i, \mathbf{z}_j \in Z$, sim denotes the cosine similarity, $\mathbf{1}_{[k \neq i]}$ denotes an indicator function evaluating to 1 iff $k \neq i$ and τ is a temperature parameter with a default value of 0.5. The final loss is computed across all positive pairs in the minibatch. To evaluate the quality of learned representations for mouse uterine benchmark, we used a linear evaluation and the nearest neighbor mining. In the linear evaluation, one average ion image was generated from each manually classified group of images as the centroid of the cluster. A linear classifier was subsequently trained on top of the frozen re-trained encoder, with 13 average ion images and their corresponding annotated labels. Next, all original ion images were classified by the encoder and the updated linear classifier. The resulting classification accuracy is used as a proxy for the quality of image representation.[6] In the nearest neighbor mining protocol, for each ion image, we searched its K nearest neighbors (K ∈ [1, 30]) based on cosine distance in the representation space. We quantified the purity of the neighborhood by counting the annotation-matching pairs for each image and their nearest neighbors. We also visualized the learned representations using t-SNE with scikit-learn default settings.

Image clustering

It has been demonstrated in a previous report[7] and this study (Fig. 2e) that images with similar high-level spatial features are mapped together in the representation space using contrastive learning. To leverage the meaningful local neighborhoods, SC was adopted to cluster ion images as the classification pretext task. Based on the cosine distance, 10 nearest neighbors of each ion image were identified to construct a graph. Next, we used the discretization approach to cluster nodes in the graph after the Laplacian embeddings.[8] After the SC, we used the resulting classification labels to initialize a learnable linear classifier on top of the CNN encoder, which enables the following self-labeling process to fine-tune the model (step 2 in Fig. 1). More specifically, the linear classifier is composed of a linear layer and a softmax function. We set the encoder in frozen mode and trained the classifier with original ion images and initial labels obtained from SC.

Self-labeling

As reported in a previous study, self-labeling improves the CNN model using a plain criterion: two independently augmented images from one ion should be classified into the same cluster. With this principle, the self-supervised training is able to enhance the generalization power of the model. In addition, only the confidently-classified ion images are included in this training.[7] As shown in Fig. 2f and Fig. S5, we observed that the classification accuracy obtained for selected mouse uterine ion images increases with an increase in the softmax probability threshold. This indicates that the softmax probability threshold may be used to exclude falsely classified ion images from the training, which enhances the accuracy of the CNN

model in the self-labeling step. In the implementation, we empirically selected the probability threshold with respect to the sample population. More specifically, after the initialization of the linear classifier, we selected the 40% quantile of softmax probabilities as the threshold. The selected training data containing 60% of the original ion images has a larger fraction of correctly classified images than the original data. Training samples were updated with the same probability threshold at every epoch, thus we gradually included more samples into the training (Fig. 2g). For each selected ion image, we applied a weak and a strong augmentations, respectively. Two pseudo labels were obtained after the encoder and classifier. A weighted cross-entropy loss was then applied to the minibatch of weakly augmented ion images to update the parameters of the CNN model.

Clustering accuracy evaluation

In this study, we used over-clustering to effectively capture the intra-class variance. The clustering accuracy was calculated using the following equation:

$$Accuracy(\mathbb{C}, \mathbb{T}) = \frac{1}{N} \sum_i \max_j |c_i \cap t_j|$$

Where $\mathbb{C} = c_1, c_2, \ldots, c_I$ is the set of predicted clusters, $\mathbb{T} = t_1, t_2, \ldots, t_J$ is the set of ground truth classes, N is the total number of ion images. In each cluster, the most frequent ground truth class was identified and assigned as a predicted class for the whole cluster. The accuracy was calculated by counting the correctly predicted ion images and dividing by N.

Comparison of the self-supervised clustering with vector-based methods

First, we evaluated the pairwise similarity measurements with different input data. In the current method, we used CNN feature vectors for the downstream classification task. For a comparative study, image vectors were generated by flattening the original images after 0.999 quantile hot spot removal as a pre-processing step. Three metrics, including Euclidean distance, cosine similarity, and Pearson correlation were calculated for comparison.

Next, we evaluated the clustering accuracy of two established machine learning methods that rely on image vectors: a density based clustering analysis with nonlinear dimensionality reduction[9] and Ward hierarchical clustering[5].The Uniform Manifold Approximation and Projection (UMAP) was performed using the umap-learn package with the following parameters: n_components = 3, n_neighbors=5, min_dist=0.5, metric = 'cosine'. We note that these parameters were optimal for visualizing and grouping of image vectors, but were not best suited for the CNN feature vectors (Fig. 2d as reference). Nevertheless, we used the same UMAP parameters in Fig. 4c and 4d for the visualization of both results for an objective comparison between them. Hierarchical Density-based Spatial Clustering for Applications with Noise (HDBSCAN) was performed to cluster UMAP representations using default parameters except for "min_cluster_size". We tuned "min_cluster_size" in order to obtain either 13 or 20 clusters. In all the HDBSCAN clustering results, the number of noise data points was less than ten. In the Ward hierarchical clustering, each vector was normalized to the range of [0, 1]. The method was implemented using scipy package (scipy.cluster.hierarchy.linkage) with the following parameters: method='ward', metric='euclidean'."

Isotopic recall evaluation

Isotopic recall is a metric reported in a previous study[10] to evaluate the performance of ion image clustering. Isotopic ion images were identified based on the *m/z* shift and Pearson correlation of the ion image with that of the candidate monoisotopic peak. Specifically, mass spectral features separated from each other by

m/z 1.003 with a tolerance of *m/z* 0.01 were assigned as candidate isotopic peaks. Second, ion images of isotopic peaks should have a Pearson correlation greater than 0.5. For the self-supervised clustering results, we counted the number of isotopic images that were correctly clustered together and divided it by the total number of identified isotopes. This fraction gives the isotopic recall, which is in the range of 0 to 1.

Data augmentation

Data augmentation is crucial for contrastive learning and self-labeling. We systematically studied the impact of data augmentation operators as listed in Table S3. For each single augmentation operation, we randomly sampled a parameter interval for an image transformation function. Moreover, we classified the compositions of data augmentation operators into three classes. Medium augmentation was used in SimCLR, while weak and strong augmentations were used in self-labeling.

Model training protocal

For SimCLR training step, we used Adam optimizer with the initial learning rate of 0.001. A cosine annealing with a period of training epochs was used to decay the learning rate. According to the previous report, SimCLR benefits from larger batch sizes.[6] In our implementation, we used the largest batch size allowed by the GPU memory and trained for 100 iterations. For the self-labeling step, an Adam optimizer with the initial learning rate of 0.0001 and the same cosine annealing schedular were used. We trained the CNN for 300 iterations. We implemented the model training on Google Colaboratory, a cloud computing platform. Using the NVIDIA Tesla P100-16GB GPU, the total training time for mouse uterine MSI benchmark and mouse brain MSI dataset was about 15 and 45 minutes, respectively.

Table S1. Information of benchmark mouse uterine dataset and unannotated mouse brain dataset.

Parameters	Benchmark dataset	Unannotated dataset
Tissue type	Mouse uterine	Mouse brain
Ionization methods	nano-DESI	MALDI
Spatial resolution (μm)	10	20
Raw pixel number	58×920	240×220
Resized pixel number	96×96	224×224
Polarity mode	Positive and negative	Positive
Mass range (*m/z*)	135-1000	600-1000
Image number	367	1101

Table S2. Accuracy of spectral clustering results with 20 clusters at conditions of varying neighborhood sizes. Ion image representations were generated by encoder shown in Fig. 2d.

**Number of neighbors**	3	5	8	10	15	20	30
**Accuracy (%)**	67.8 ± 2.4	92.3 ± 1.0	92.8 ± 0.1	92.2 ± 0.3	90.8 ± 0.7	88.8 ± 2.1	85.8 ± 0.8

Table S3. A summary of image augmentation operator parameters. The kernel size in Gaussian blur is set to be 10% of the image height/width. Off-tissue mask was not applied to unannotated dataset.

Augmentation class	Transformation	Parameter	Interval
Weak	Gaussian blur	sigma	[0.001, 0.4]
	Gaussian noise	sigma	[0.001, 0.1]
	Color Jitter	brightness, contrast, saturation, hue	0.25, 0.25, 0.25, 0.2
	Off-tissue mask	N/A	N/A
Medium	Gaussian blur	sigma	[0.01, 0.75]
	Gaussian noise	sigma	[0.001, 0.2]
	Color Jitter	brightness, contrast, saturation, hue	0.5, 0.5, 0.5, 0.2
Strong	Gaussian blur	sigma	[0.1, 2]
	Gaussian noise	sigma	[0.001, 0.4]
	Color Jitter	brightness, contrast, saturation, hue	0.5, 0.5, 0.5, 0.2
	Off-tissue mask	N/A	N/A

Table S4. Comparison of the classification accuracy of different image vector-based clustering methods on benchmark data.

Clustering method	Number of clusters	Accuracy (%)
Ward Hierarchical Clustering	13	70.2
UMAP + HDBSCAN	13	58.3 ± 2.1
Self-Supervised Clustering	13	**84.0 ± 3.1**
Ward Hierarchical Clustering	20	80.9
UMAP + HDBSCAN	20	68.4 ± 2.0
Self-Supervised Clustering	20	**92.7 ± 2.1**

Table S5. A variety of pairwise similarity measurements for image vector and CNN feature vectors.

Group	Reference ion	Other ion	Image vector			CNN feature vector		
			Euclidean distance	Cosine similarity	Pearson similarity	Euclidean distance	Cosine similarity	Pearson similarity
1	pos, 875.5700	pos, 739.4681	35.5	0.720	0.609	23.2	0.420	0.406
		pos, 868.5243	38.7	0.685	0.564	**18.6**	**0.695**	**0.687**
2	neg, 471.2616	neg, 684.6076	23.5	0.745	0.724	25.6	0.626	0.560
		pos, 793.5930	35.7	0.595	0.553	**18.4**	**0.785**	**0.759**
3	pos, 820.6181	pos, 594.3172	32.8	0.596	0.552	22.0	0.530	0.496
		pos, 707.5007	33.7	0.563	0.521	**10.3**	**0.873**	**0.865**
4	neg, 293.5339	neg, 309.5065	44.3	0.573	0.466	32.8	0.232	0.186
		pos, 838.5600	47.6	0.513	0.398	**27.5**	**0.420**	**0.399**

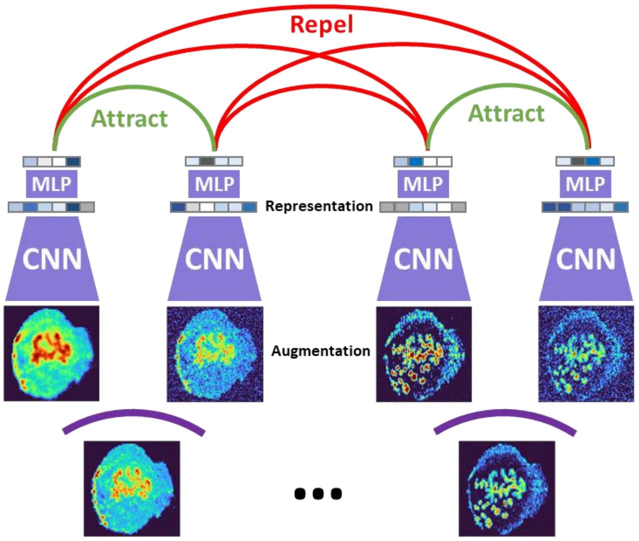

Figure S1. The framework of SimCLR.

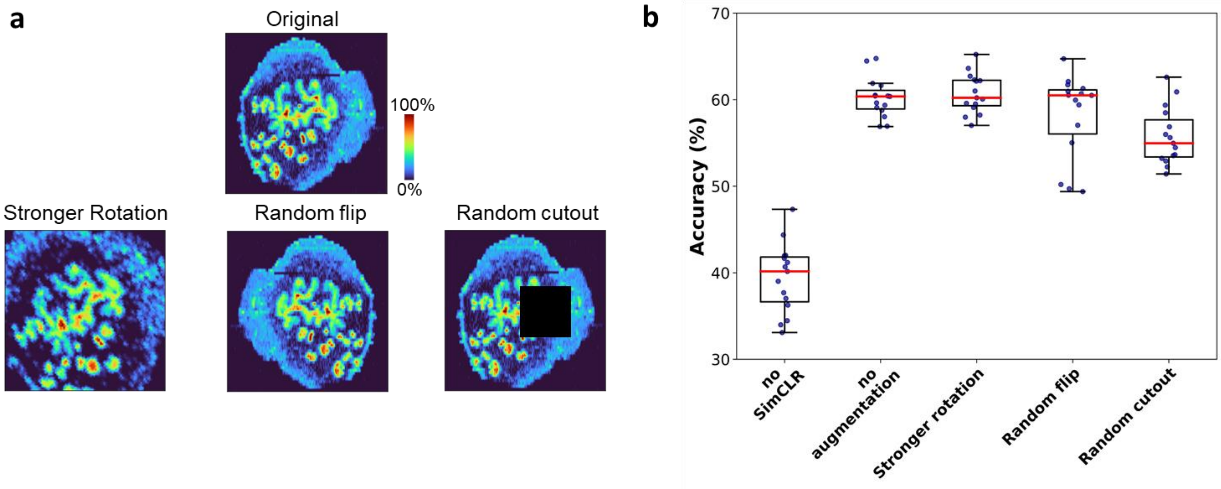

Figure S2. Contrastive learning with strong geometry-changing augmentations. (a) Illustration of strong geometry-changing augmentations used in current study. (b) Linear evaluation of re-trained CNN encoder with individual augmentation shown in panel a. Results of transfer learning and SimCLR without data augmentation are also listed for comparison.

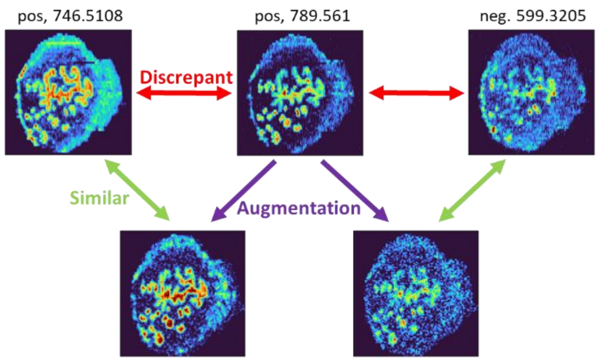

Figure S3. Generalization power provided by composite image augmentation strategy.

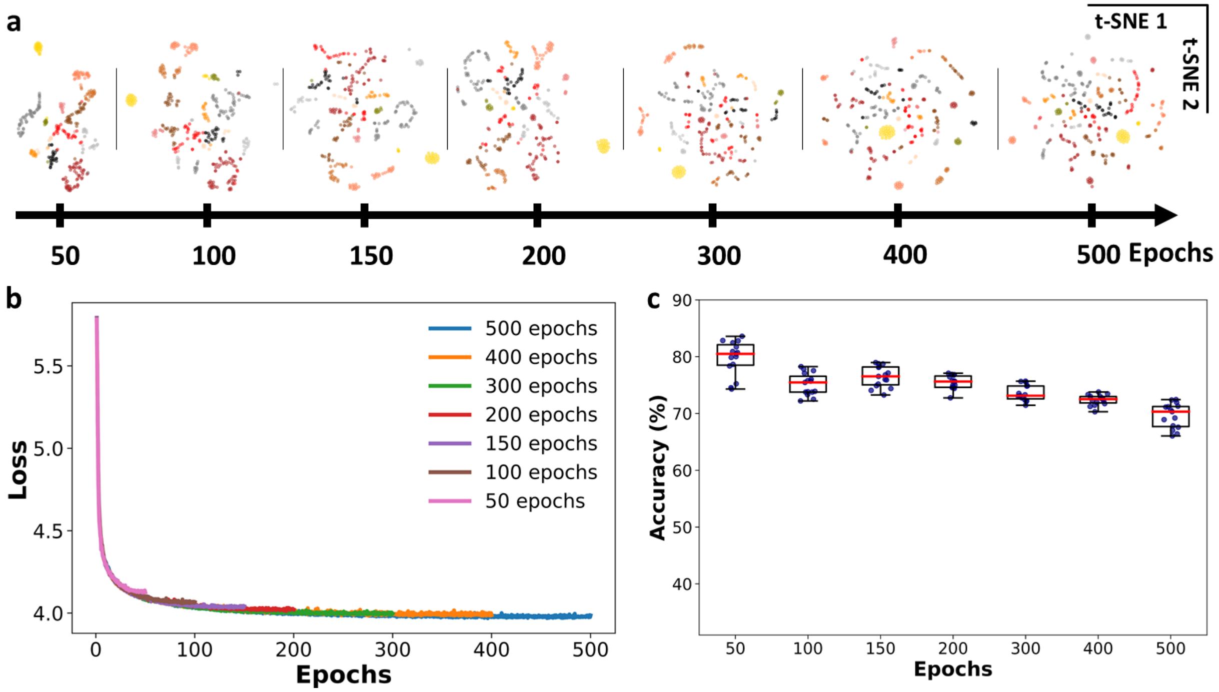

Figure S4. The impact of training time on learned image representations during contrastive learning. (a) t-SNE visualizations of learned representations with different training epochs. (b) Contrastive losses during trainings with different epoch settings. (c) Linear evaluation of re-trained CNN encoder with different epoch settings.

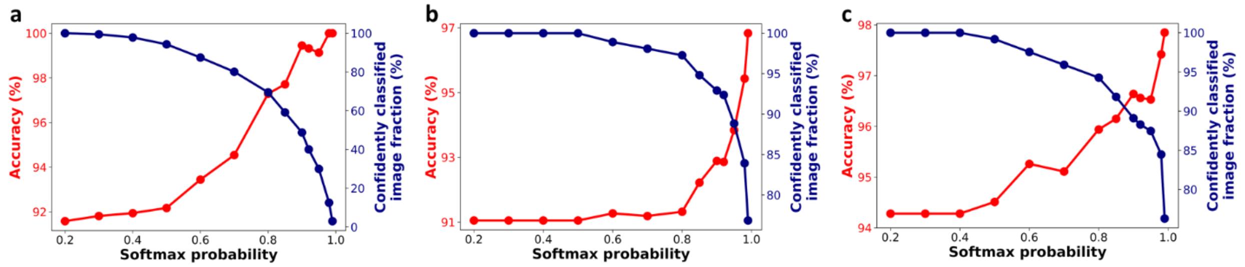

Figure S5. Relationships between classification accuracy and confidently classified image fraction obtained from (a) CNN model which was trained by initial labels and classified ion images into 20 groups. (b) CNN model which was trained by self-labeling and classified ion images into 13 groups and (c) CNN model which was trained by self-labeling and classified ion images into 20 groups. Each data point was calculated from an ion image subset which was selected by a softmax probability threshold.

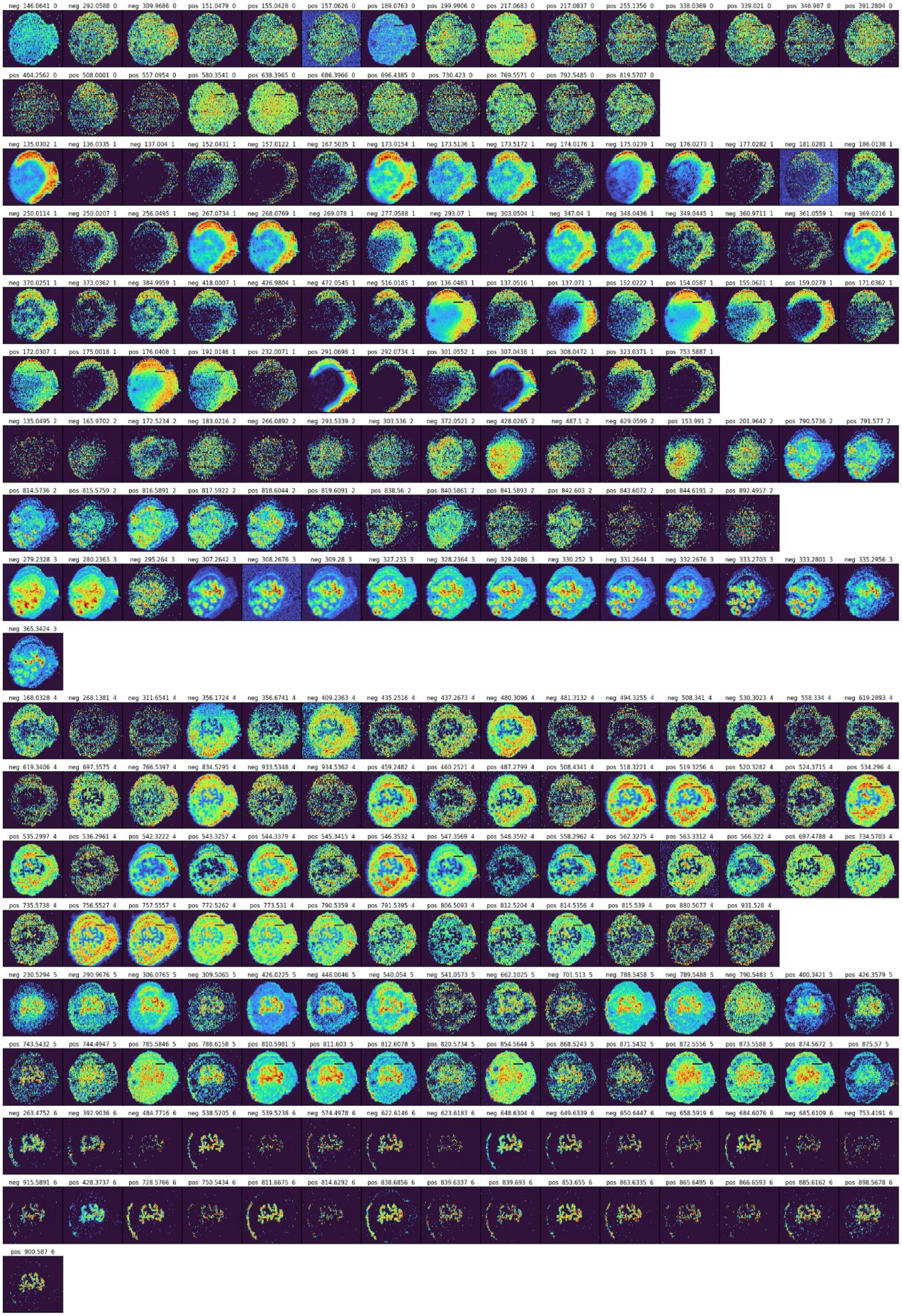
neg 146.0641 0
neg 292.0588 0
neg 309.9686 0
pos 151.0479 0
pos 155.0428 0
pos 157.0626 0
pos 189.0763 0
pos 199.9906 0
pos 217.0683 0
pos 217.0837 0
pos 255.1356 0
pos 338.0369 0
pos 339.021 0
pos 346.987 0
pos 391.2804 0
pos 404.2562 0
pos 508.0001 0
pos 557.0954 0
pos 580.3541 0
pos 638.3965 0
pos 686.3966 0
pos 696.4385 0
pos 730.423 0
pos 769.5571 0
pos 792.5485 0
pos 819.5707 0
neg 135.0302 1
neg 136.0335 1
neg 137.004 1
neg 152.0431 1
neg 157.0122 1
neg 167.5035 1
neg 173.0154 1
neg 173.5136 1
neg 173.5172 1
neg 174.0176 1
neg 175.0239 1
neg 176.0273 1
neg 177.0282 1
neg 181.0281 1
neg 186.0138 1
neg 250.0114 1
neg 250.0207 1
neg 256.0495 1
neg 267.0734 1
neg 268.0769 1
neg 269.078 1
neg 277.0588 1
neg 293.07 1
neg 303.0504 1
neg 347.04 1
neg 348.0436 1
neg 349.0445 1
neg 360.9711 1
neg 361.0559 1
neg 369.0216 1
neg 370.0251 1
neg 373.0362 1
neg 384.9959 1
neg 418.0007 1
neg 426.9804 1
neg 472.0545 1
neg 516.0185 1
pos 136.0483 1
pos 137.0516 1
pos 137.071 1
pos 152.0222 1
pos 154.0587 1
pos 155.0621 1
pos 159.0278 1
pos 171.0362 1
pos 172.0307 1
pos 175.0018 1
pos 176.0408 1
pos 192.0146 1
pos 232.0071 1
pos 291.0698 1
pos 292.0734 1
pos 301.0552 1
pos 307.0438 1
pos 308.0472 1
pos 323.0371 1
pos 753.5887 1
neg 135.0495 2
neg 165.9702 2
neg 172.5234 2
neg 183.0216 2
neg 266.0892 2
neg 293.5339 2
neg 303.536 2
neg 372.0521 2
neg 428.0265 2
neg 487.1 2
neg 629.0599 2
pos 153.991 2
pos 201.9642 2
pos 790.5736 2
pos 791.577 2
pos 814.5736 2
pos 815.5759 2
pos 816.5891 2
pos 817.5922 2
pos 818.6044 2
pos 819.6091 2
pos 838.56 2
pos 840.5861 2
pos 841.5893 2
pos 842.603 2
pos 843.6072 2
pos 844.6191 2
pos 892.4957 2
neg 279.2328 3
neg 280.2363 3
neg 295.264 3
neg 307.2642 3
neg 308.2676 3
neg 309.28 3
neg 327.233 3
neg 328.2364 3
neg 329.2486 3
neg 330.252 3
neg 331.2644 3
neg 332.2676 3
neg 333.2703 3
neg 333.2801 3
neg 335.2956 3
neg 365.3424 3
neg 168.0328 4
neg 268.1381 4
neg 311.6541 4
neg 356.1724 4
neg 356.6741 4
neg 409.2363 4
neg 435.2516 4
neg 437.2673 4
neg 480.3096 4
neg 481.3132 4
neg 494.3255 4
neg 508.341 4
neg 530.3023 4
neg 558.334 4
neg 619.2893 4
neg 619.3406 4
neg 697.3575 4
neg 766.5397 4
neg 834.5295 4
neg 933.5348 4
neg 934.5362 4
pos 459.2482 4
pos 460.2521 4
pos 487.2799 4
pos 508.4341 4
pos 518.3221 4
pos 519.3256 4
pos 520.3282 4
pos 524.3715 4
pos 534.296 4
pos 535.2997 4
pos 536.2961 4
pos 542.3222 4
pos 543.3257 4
pos 544.3379 4
pos 545.3415 4
pos 546.3532 4
pos 547.3569 4
pos 548.3592 4
pos 558.2962 4
pos 562.3275 4
pos 563.3312 4
pos 566.322 4
pos 697.4788 4
pos 734.5703 4
pos 735.5738 4
pos 756.5527 4
pos 757.5557 4
pos 772.5262 4
pos 773.531 4
pos 790.5359 4
pos 791.5395 4
pos 806.5093 4
pos 812.5204 4
pos 814.5356 4
pos 815.539 4
pos 880.5077 4
pos 931.528 4
neg 230.5294 5
neg 290.9676 5
neg 306.0765 5
neg 309.5065 5
neg 426.0225 5
neg 448.0046 5
neg 540.054 5
neg 541.0573 5
neg 662.1025 5
neg 701.513 5
neg 788.5458 5
neg 789.5488 5
neg 790.5483 5
pos 400.3421 5
pos 426.3579 5
pos 743.5432 5
pos 744.4947 5
pos 785.5846 5
pos 788.6158 5
pos 810.5981 5
pos 811.603 5
pos 812.6078 5
pos 820.5734 5
pos 854.5644 5
pos 868.5243 5
pos 871.5432 5
pos 872.5556 5
pos 873.5588 5
pos 874.5672 5
pos 875.57 5
neg 263.4752 6
neg 392.9036 6
neg 484.7716 6
neg 538.5205 6
neg 539.5238 6
neg 574.4978 6
neg 622.6146 6
neg 623.6183 6
neg 648.6304 6
neg 649.6339 6
neg 650.6447 6
neg 658.5919 6
neg 684.6076 6
neg 685.6109 6
neg 753.4191 6
neg 915.5891 6
pos 428.3737 6
pos 728.5766 6
pos 750.5434 6
pos 811.6675 6
pos 814.6292 6
pos 838.6856 6
pos 839.6337 6
pos 839.693 6
pos 853.655 6
pos 863.6335 6
pos 865.6495 6
pos 866.6593 6
pos 885.6162 6
pos 898.5678 6
pos 900.587 6

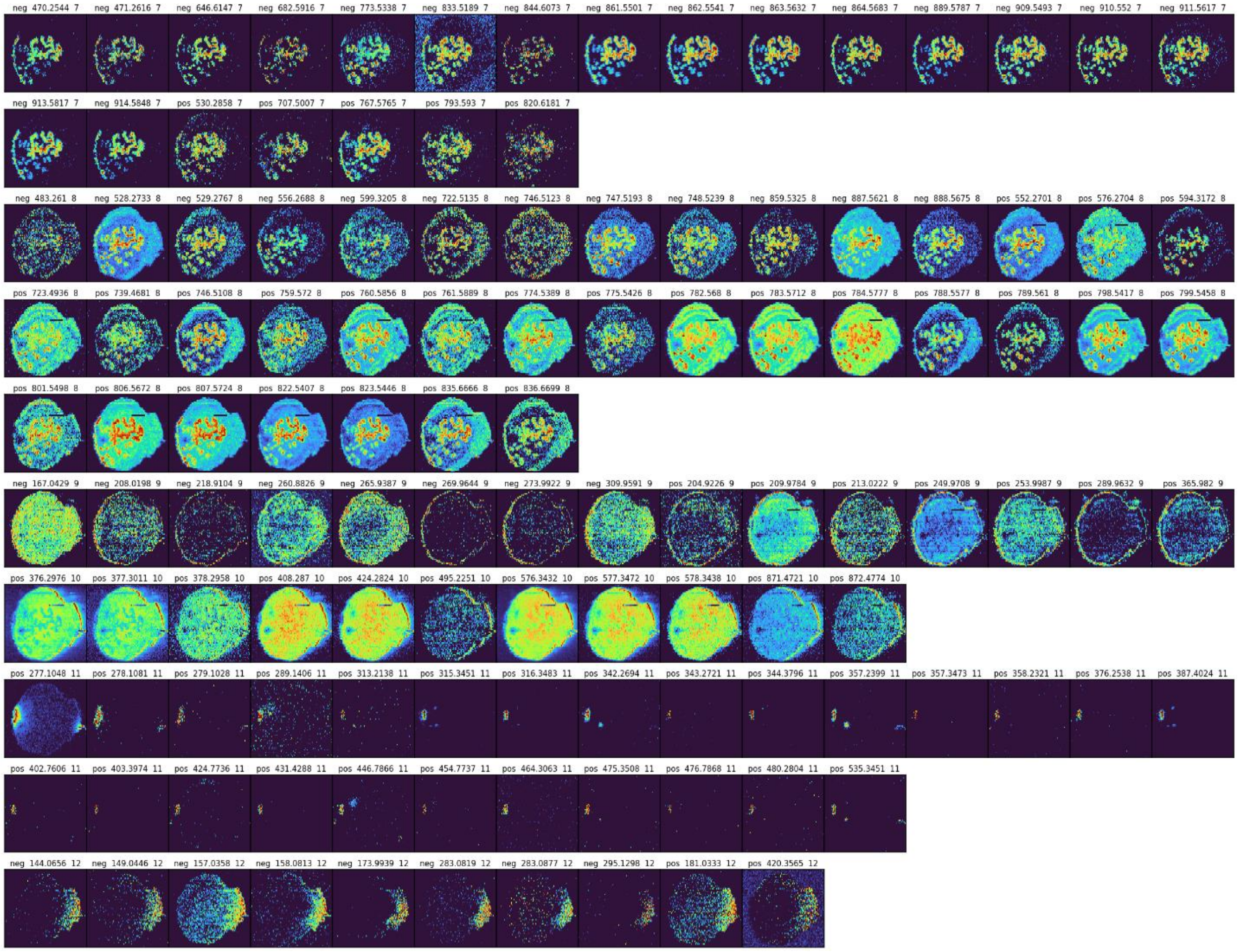

Figure S6. Manual classification of 367 ion images in the mouse uterine benchmark.

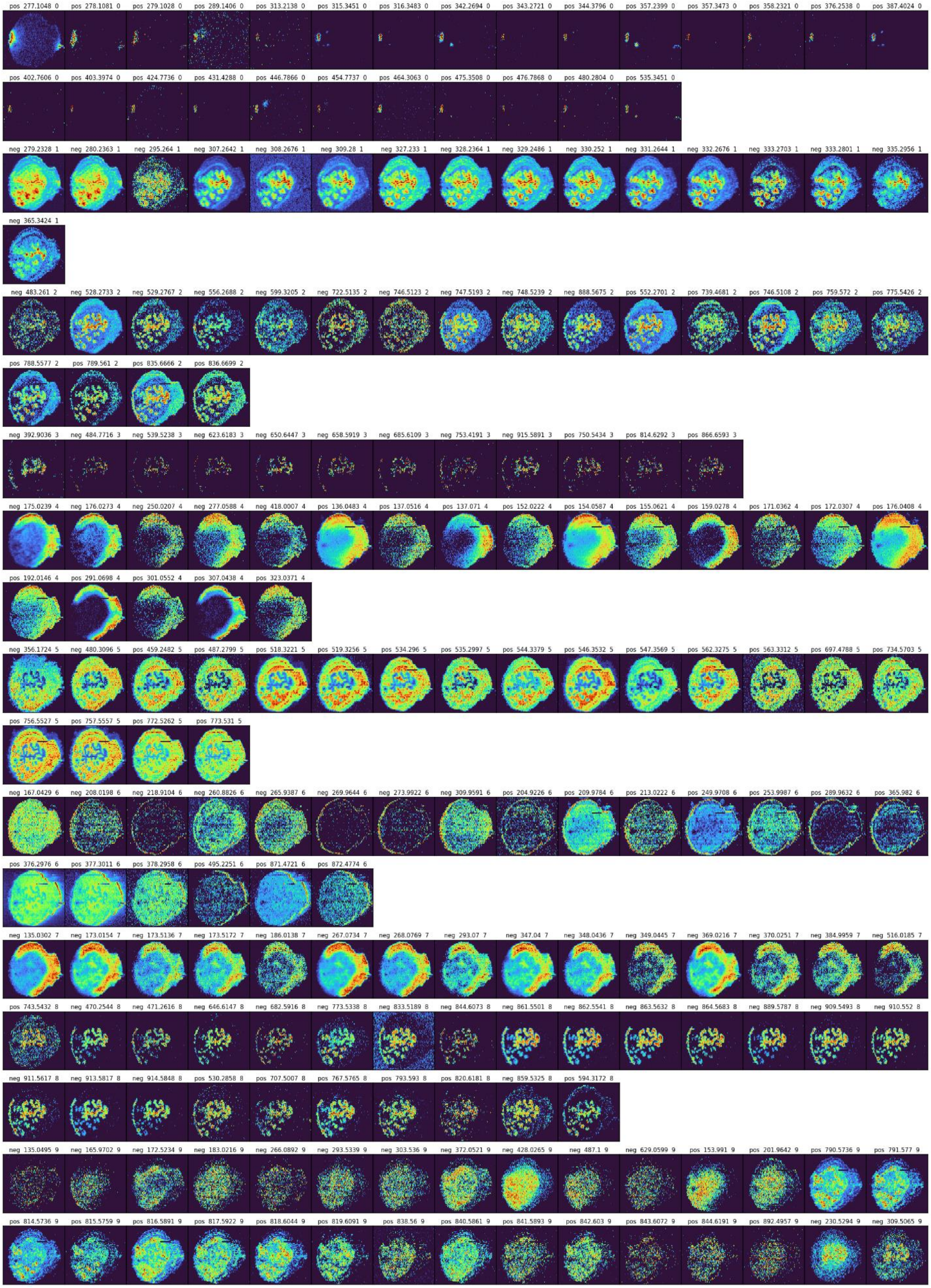
pos 277.1048 0
pos 278.1081 0
pos 279.1028 0
pos 289.1406 0
pos 313.2138 0
pos 315.3451 0
pos 316.3483 0
pos 342.2694 0
pos 343.2721 0
pos 344.3796 0
pos 357.2399 0
pos 357.3473 0
pos 358.2321 0
pos 376.2538 0
pos 387.4024 0
pos 402.7606 0
pos 403.3974 0
pos 424.7736 0
pos 431.4288 0
pos 446.7866 0
pos 454.7737 0
pos 464.3063 0
pos 475.3508 0
pos 476.7868 0
pos 480.2804 0
pos 535.3451 0
neg 279.2328 1
neg 280.2363 1
neg 295.264 1
neg 307.2642 1
neg 308.2676 1
neg 309.28 1
neg 327.233 1
neg 328.2364 1
neg 329.2486 1
neg 330.252 1
neg 331.2644 1
neg 332.2676 1
neg 333.2703 1
neg 333.2801 1
neg 335.2956 1
neg 365.3424 1
neg 483.261 2
neg 528.2733 2
neg 529.2767 2
neg 556.2688 2
neg 599.3205 2
neg 722.5135 2
neg 746.5123 2
neg 747.5193 2
neg 748.5239 2
neg 888.5675 2
pos 552.2701 2
pos 739.4681 2
pos 746.5108 2
pos 759.572 2
pos 775.5426 2
pos 788.5577 2
pos 789.561 2
pos 835.6666 2
pos 836.6699 2
neg 392.9036 3
neg 484.7716 3
neg 539.5238 3
neg 623.6183 3
neg 650.6447 3
neg 658.5919 3
neg 685.6109 3
neg 753.4191 3
neg 915.5891 3
pos 750.5434 3
pos 814.6292 3
pos 866.6593 3
neg 175.0239 4
neg 176.0273 4
neg 250.0207 4
neg 277.0588 4
neg 418.0007 4
pos 136.0483 4
pos 137.0516 4
pos 137.071 4
pos 152.0222 4
pos 154.0587 4
pos 155.0621 4
pos 159.0278 4
pos 171.0362 4
pos 172.0307 4
pos 176.0408 4
pos 192.0146 4
pos 291.0698 4
pos 301.0552 4
pos 307.0438 4
pos 323.0371 4
neg 356.1724 5
neg 480.3096 5
pos 459.2482 5
pos 487.2799 5
pos 518.3221 5
pos 519.3256 5
pos 534.296 5
pos 535.2997 5
pos 544.3379 5
pos 546.3532 5
pos 547.3569 5
pos 562.3275 5
pos 563.3312 5
pos 697.4788 5
pos 734.5703 5
pos 756.5527 5
pos 757.5557 5
pos 772.5262 5
pos 773.531 5
neg 167.0429 6
neg 208.0198 6
neg 218.9104 6
neg 260.8826 6
neg 265.9387 6
neg 269.9644 6
neg 273.9922 6
neg 309.9591 6
pos 204.9226 6
pos 209.9784 6
pos 213.0222 6
pos 249.9708 6
pos 253.9987 6
pos 289.9632 6
pos 365.982 6
pos 376.2976 6
pos 377.3011 6
pos 378.2958 6
pos 495.2251 6
pos 871.4721 6
pos 872.4774 6
neg 135.0302 7
neg 173.0154 7
neg 173.5136 7
neg 173.5172 7
neg 186.0138 7
neg 267.0734 7
neg 268.0769 7
neg 293.07 7
neg 347.04 7
neg 348.0436 7
neg 349.0445 7
neg 369.0216 7
neg 370.0251 7
neg 384.9959 7
neg 516.0185 7
pos 743.5432 8
pos 470.2544 8
pos 471.2616 8
pos 646.6147 8
pos 682.5916 8
pos 773.5338 8
pos 833.5189 8
neg 844.6073 8
neg 861.5501 8
neg 862.5541 8
neg 863.5632 8
neg 864.5683 8
neg 889.5787 8
neg 909.5493 8
neg 910.552 8
neg 911.5617 8
neg 913.5817 8
neg 914.5848 8
pos 530.2858 8
pos 707.5007 8
pos 767.5765 8
pos 793.593 8
pos 820.6181 8
neg 859.5325 8
pos 594.3172 8
neg 135.0495 9
neg 165.9702 9
neg 172.5234 9
neg 183.0216 9
neg 266.0892 9
neg 293.5339 9
neg 303.536 9
neg 372.0521 9
neg 428.0265 9
neg 487.1 9
neg 629.0599 9
pos 153.991 9
pos 201.9642 9
pos 790.5736 9
pos 791.577 9
pos 814.5736 9
pos 815.5759 9
pos 816.5891 9
pos 817.5922 9
pos 818.6044 9
pos 819.6091 9
pos 838.56 9
pos 840.5861 9
pos 841.5893 9
pos 842.603 9
pos 843.6072 9
pos 844.6191 9
pos 892.4957 9
neg 230.5294 9
neg 309.5065 9

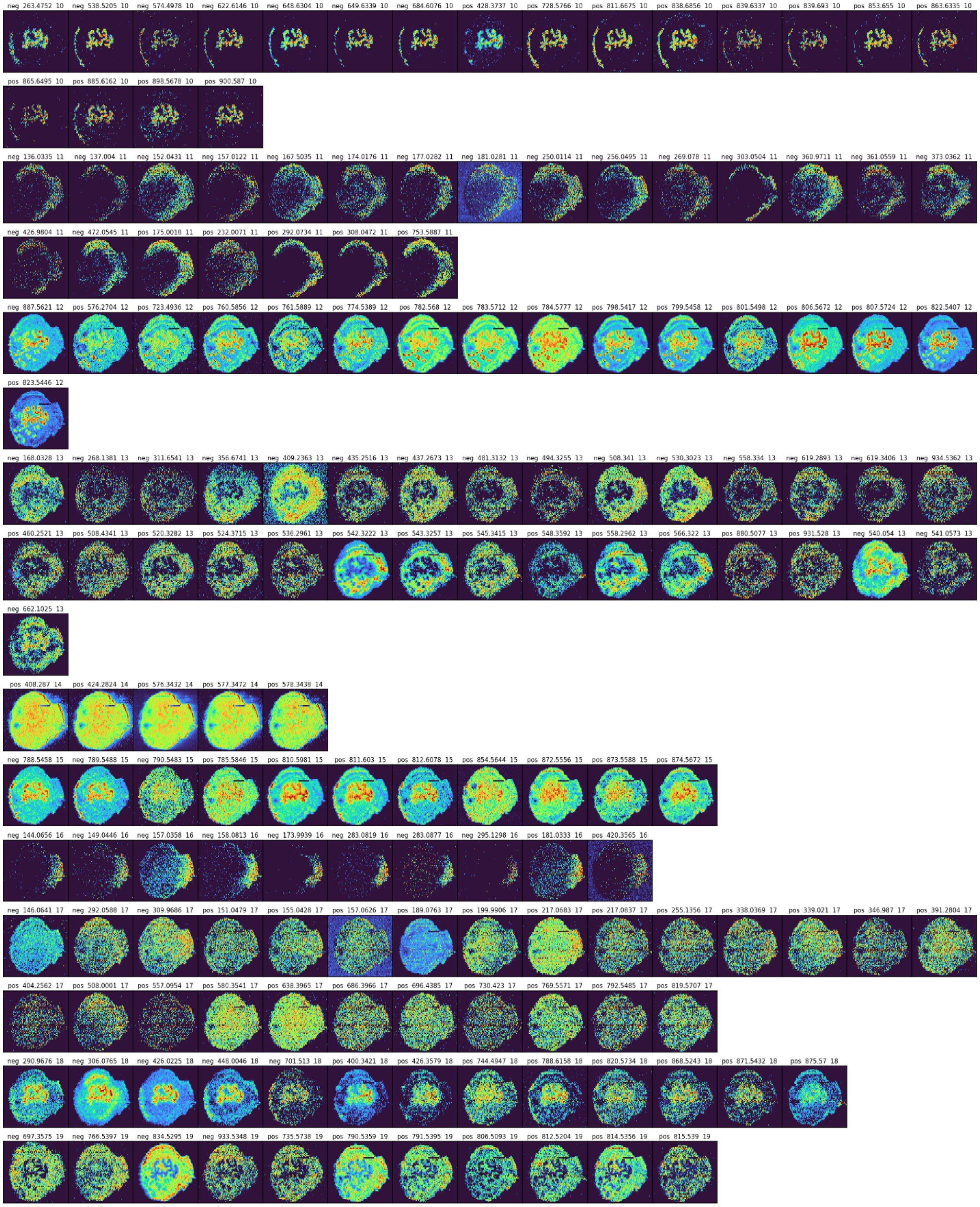

Figure S7. Self-supervised clustering results of 367 ion images in the mouse uterine benchmark.

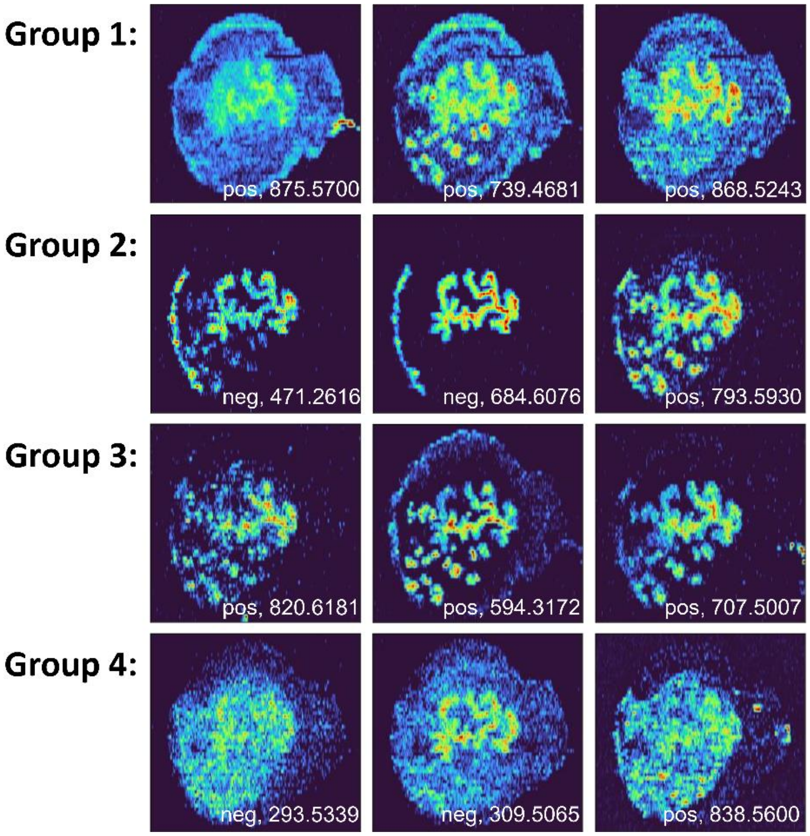

Figure S8. Four groups of ion images for pairwise similarity measurement discussion.

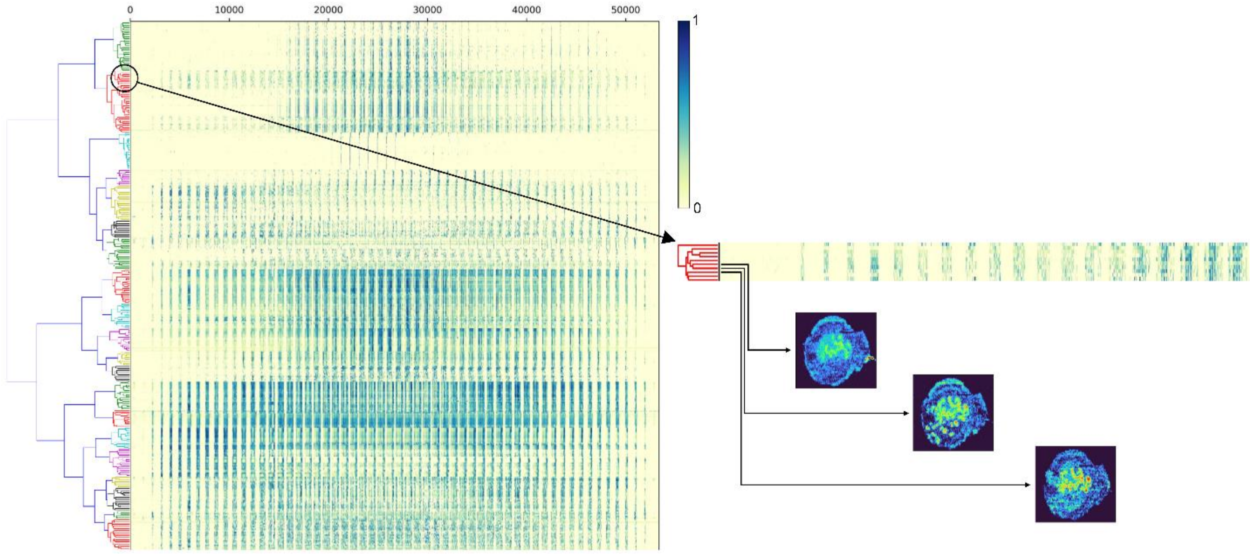

Figure S9. Dendrogram and sorted image vectors in Ward hierarchical clustering results. A zoom-in image shows that three ion images (shown in Fig. 4a) are clustered together. Although Ward hierarchical clustering is able to capture major patterns in input data, it cannot differentiate between the ion image of *m/z* 739.4681 and two other ion images used in this example.

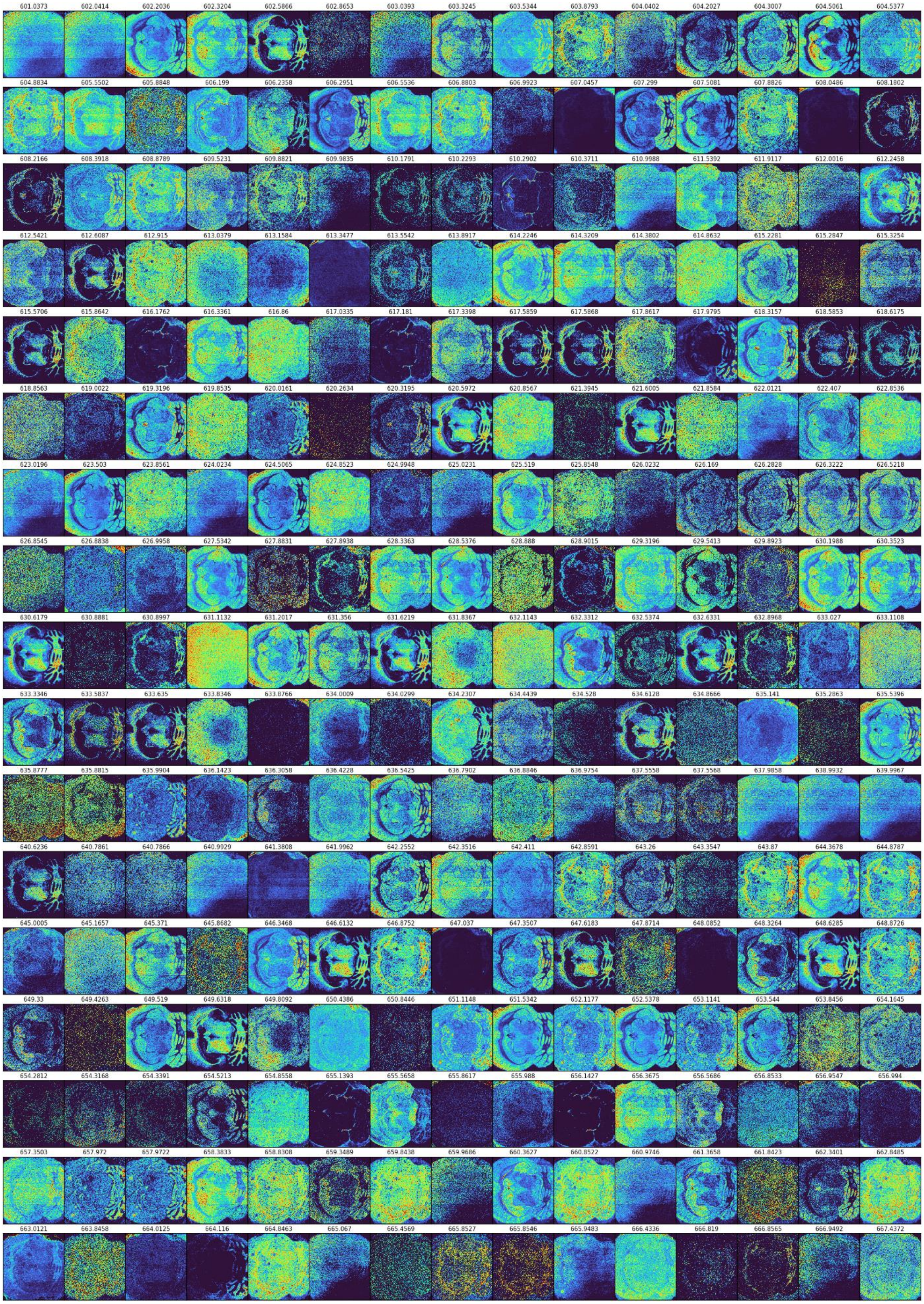
601.0373
602.0414
602.2036
602.3204
602.5866
602.8653
603.0393
603.3245
603.5344
603.8793
604.0402
604.2027
604.3007
604.5061
604.5377
604.8834
605.5502
605.8848
606.199
606.2358
606.2951
606.5536
606.8803
606.9923
607.0457
607.299
607.5081
607.8826
608.0486
608.1802
608.2166
608.3918
608.8789
609.5231
609.8821
609.9835
610.1791
610.2293
610.2902
610.3711
610.9988
611.5392
611.9117
612.0016
612.2458
612.5421
612.6087
612.915
613.0379
613.1584
613.3477
613.5542
613.8917
614.2246
614.3209
614.3802
614.8632
615.2281
615.2847
615.3254
615.5706
615.8642
616.1762
616.3361
616.86
617.0335
617.181
617.3398
617.5859
617.5868
617.8617
617.9795
618.3157
618.5853
618.6175
618.8563
619.0022
619.3196
619.8535
620.0161
620.2634
620.3195
620.5972
620.8567
621.3945
621.6005
621.8584
622.0121
622.407
622.8536
623.0196
623.503
623.8561
624.0234
624.5065
624.8523
624.9948
625.0231
625.519
625.8548
626.0232
626.169
626.2828
626.3222
626.5218
626.8545
626.8838
626.9958
627.5342
627.8831
627.8938
628.3363
628.5376
628.888
628.9015
629.3196
629.5413
629.8923
630.1988
630.3523
630.6179
630.8881
630.8997
631.1132
631.2017
631.356
631.6219
631.8367
632.1143
632.3312
632.5374
632.6331
632.8968
633.027
633.1108
633.3346
633.5837
633.635
633.8346
633.8766
634.0009
634.0299
634.2307
634.4439
634.528
634.6128
634.8666
635.141
635.2863
635.5396
635.8777
635.8815
635.9904
636.1423
636.3058
636.4228
636.5425
636.7902
636.8846
636.9754
637.5558
637.5568
637.9858
638.9932
639.9967
640.6236
640.7861
640.7866
640.9929
641.3808
641.9962
642.2552
642.3516
642.411
642.8591
643.26
643.3547
643.87
644.3678
644.8787
645.0005
645.1657
645.371
645.8682
646.3468
646.6132
646.8752
647.037
647.3507
647.6183
647.8714
648.0852
648.3264
648.6285
648.8726
649.33
649.4263
649.519
649.6318
649.8092
650.4386
650.8446
651.1148
651.5342
652.1177
652.5378
653.1141
653.544
653.8456
654.1645
654.2812
654.3168
654.3391
654.5213
654.8558
655.1393
655.5658
655.8617
655.988
656.1427
656.3675
656.5686
656.8533
656.9547
656.994
657.3503
657.972
657.9722
658.3833
658.8308
659.3489
659.8438
659.9686
660.3627
660.8522
660.9746
661.3658
661.8423
662.3401
662.8485
663.0121
663.8458
664.0125
664.116
664.8463
665.067
665.4569
665.8527
665.8546
665.9483
666.4336
666.819
666.8565
666.9492
667.4372

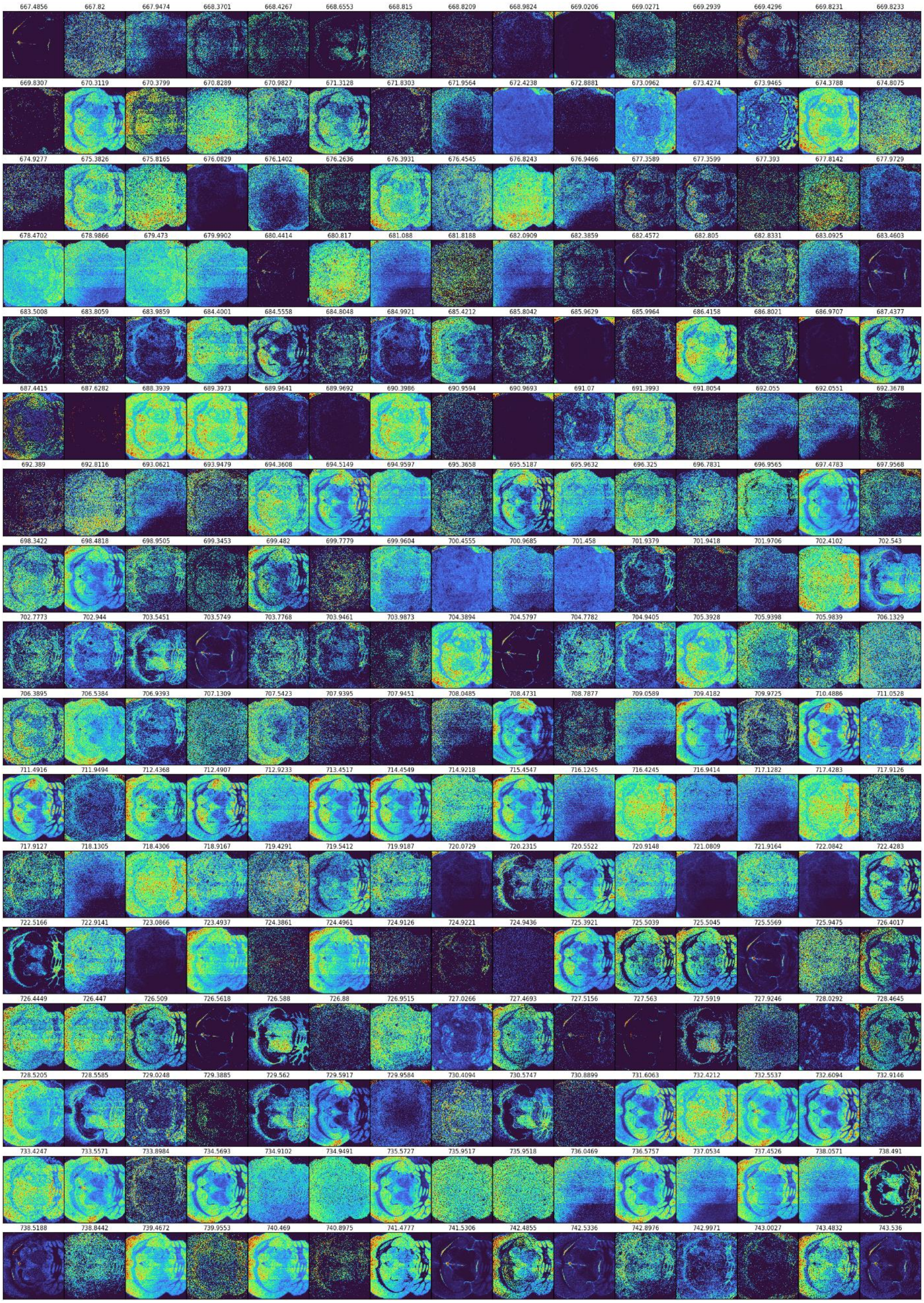

667.4856 667.82 667.9474 668.3701 668.4267 668.6553 668.815 668.8209 668.9824 669.0206 669.0271 669.2939 669.4296 669.8231 669.8233
669.8307 670.3119 670.3799 670.8289 670.9827 671.3128 671.8303 671.9564 672.4238 672.8881 673.0962 673.4274 673.9465 674.3788 674.8075
674.9277 675.3826 675.8165 676.0829 676.1402 676.2636 676.3931 676.4545 676.8243 676.9466 677.3589 677.3599 677.393 677.8142 677.9729
678.4702 678.9866 679.473 679.9902 680.4414 680.817 681.088 681.8188 682.0909 682.3859 682.4572 682.805 682.8331 683.0925 683.4603
683.5008 683.8059 683.9859 684.4001 684.5558 684.8048 684.9921 685.4212 685.8042 685.9629 685.9964 686.4158 686.8021 686.9707 687.4377
687.4415 687.6282 688.3939 689.3973 689.9641 689.9692 690.3986 690.9594 690.9693 691.07 691.3993 691.8054 692.055 692.0551 692.3678
692.389 692.8116 693.0621 693.9479 694.3608 694.5149 694.9597 695.3658 695.5187 695.9632 696.325 696.7831 696.9565 697.4783 697.9568
698.3422 698.4818 698.9505 699.3453 699.482 699.7779 699.9604 700.4555 700.9685 701.458 701.9379 701.9418 701.9706 702.4102 702.543
702.7773 702.944 703.5451 703.5749 703.7768 703.9461 703.9873 704.3894 704.5797 704.7782 704.9405 705.3928 705.9398 705.9839 706.1329
706.3895 706.5384 706.9393 707.1309 707.5423 707.9395 707.9451 708.0485 708.4731 708.7877 709.0589 709.4182 709.9725 710.4886 711.0528
711.4916 711.9494 712.4368 712.4907 712.9233 713.4517 714.4549 714.9218 715.4547 716.1245 716.4245 716.9414 717.1282 717.4283 717.9126
717.9127 718.1305 718.4306 718.9167 719.4291 719.5412 719.9187 720.0729 720.2315 720.5522 720.9148 721.0809 721.9164 722.0842 722.4283
722.5166 722.9141 723.0866 723.4937 724.3861 724.4961 724.9126 724.9221 724.9436 725.3921 725.5039 725.5045 725.5569 725.9475 726.4017
726.4449 726.447 726.509 726.5618 726.588 726.88 726.9515 727.0266 727.4693 727.5156 727.563 727.5919 727.9246 728.0292 728.4645
728.5205 728.5585 729.0248 729.3885 729.562 729.5917 729.9584 730.4094 730.5747 730.8899 731.6063 732.4212 732.5537 732.6094 732.9146
733.4247 733.5571 733.8984 734.5693 734.9102 734.9491 735.5727 735.9517 735.9518 736.0469 736.5757 737.0534 737.4526 738.0571 738.491
738.5188 738.8442 739.4672 739.9553 740.469 740.8975 741.4777 741.5306 742.4855 742.5336 742.8976 742.9971 743.0027 743.4832 743.536

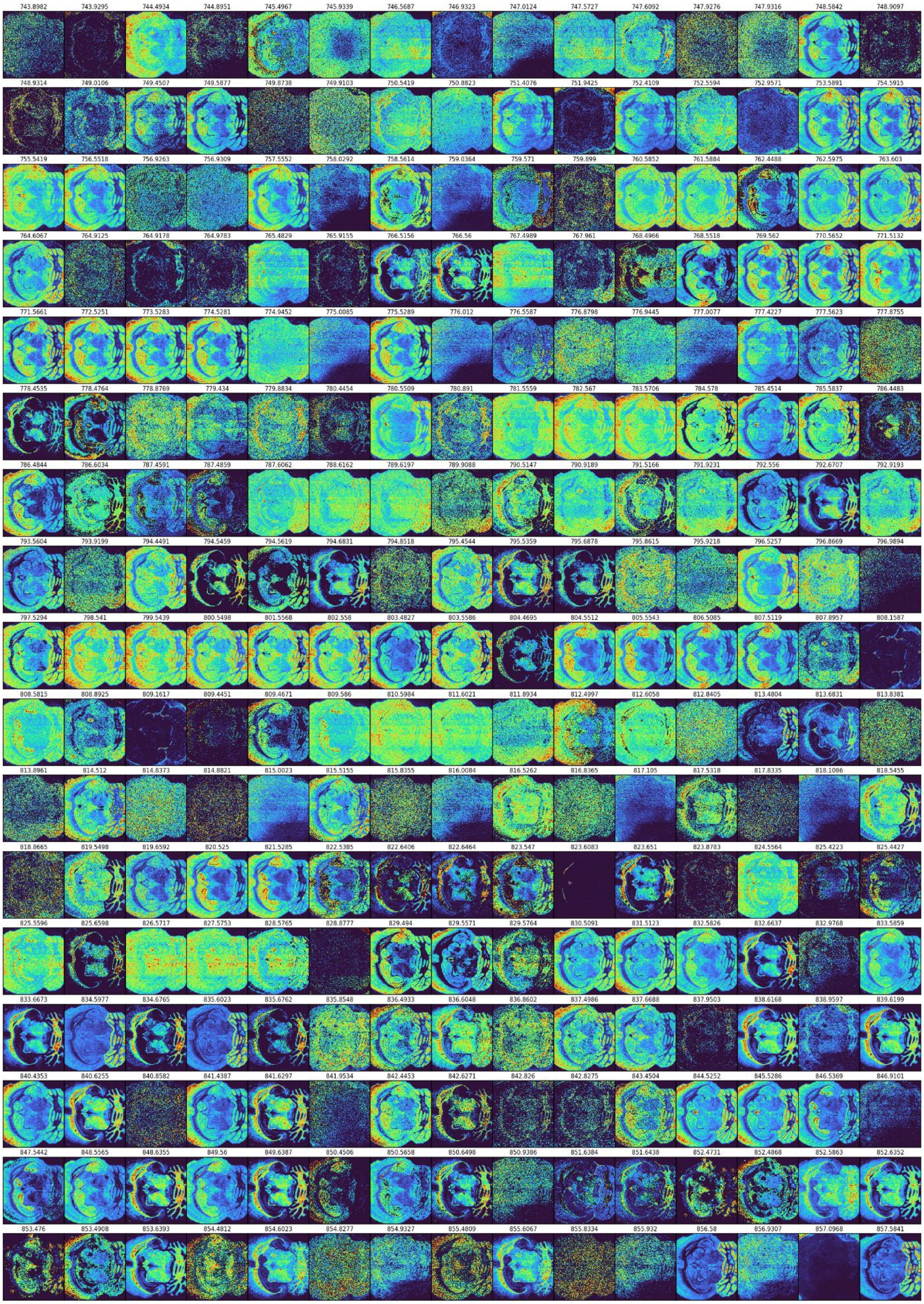

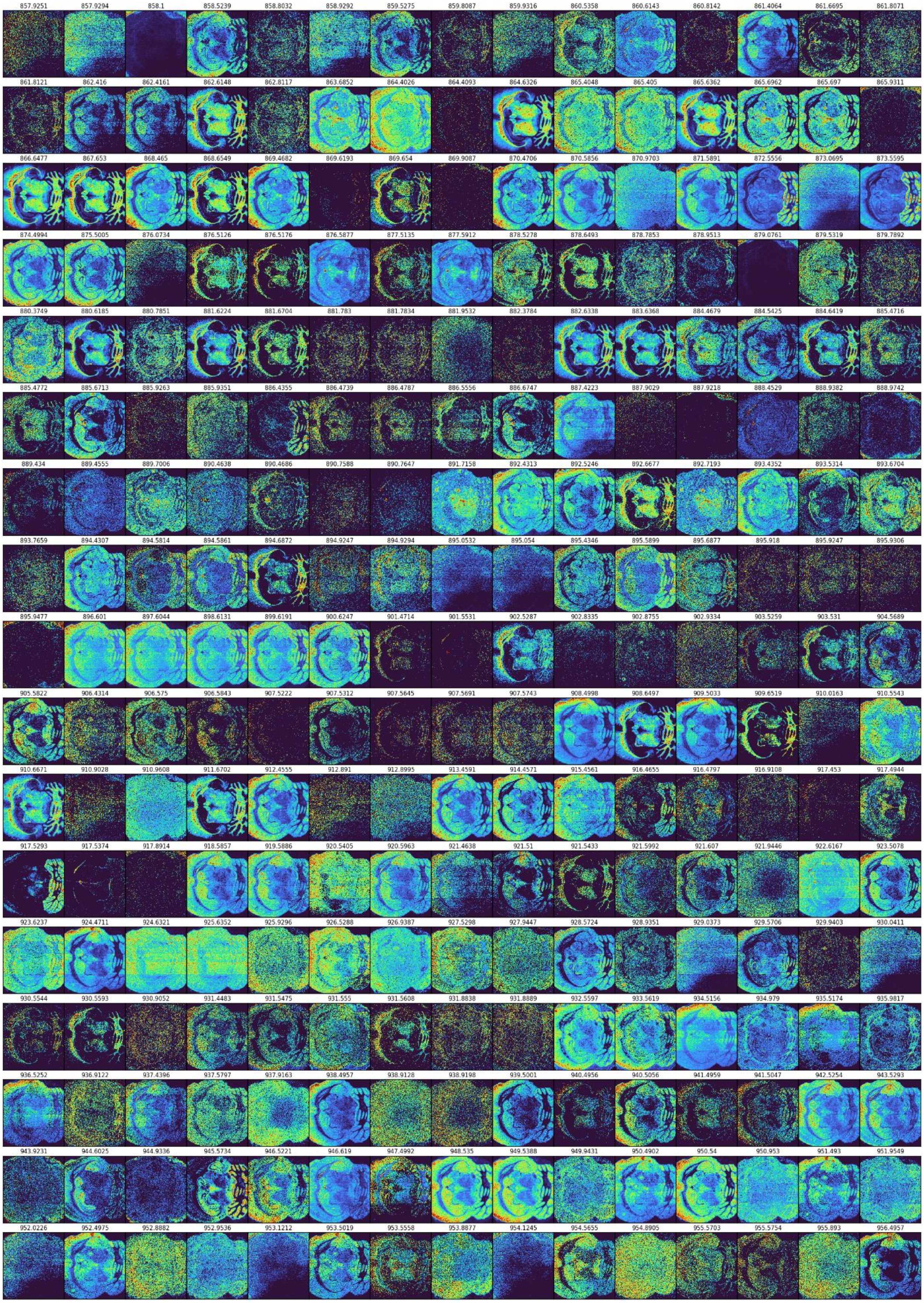
857.9251 857.9294 858.1 858.5239 858.8032 858.9292 859.5275 859.8087 859.9316 860.5358 860.6143 860.8142 861.4064 861.6695 861.8071
861.8121 862.416 862.4161 862.6148 862.8117 863.6852 864.4026 864.4093 864.6326 865.4048 865.405 865.6362 865.6962 865.697 865.9311
866.6477 867.653 868.465 868.6549 869.4682 869.6193 869.654 869.9087 870.4706 870.5856 870.9703 871.5891 872.5556 873.0695 873.5595
874.4994 875.5005 876.0734 876.5126 876.5176 876.5877 877.5135 877.5912 878.5278 878.6493 878.7853 878.9513 879.0761 879.5319 879.7892
880.3749 880.6185 880.7851 881.6224 881.6704 881.783 881.7834 881.9532 882.3784 882.6338 883.6368 884.4679 884.5425 884.6419 885.4716
885.4772 885.6713 885.9263 885.9351 886.4355 886.4739 886.4787 886.5556 886.6747 887.4223 887.9029 887.9218 888.4529 888.9382 888.9742
889.434 889.4555 889.7006 890.4638 890.4686 890.7588 890.7647 891.7158 892.4313 892.5246 892.6677 892.7193 893.4352 893.5314 893.6704
893.7659 894.4307 894.5814 894.5861 894.6872 894.9247 894.9294 895.0532 895.054 895.4346 895.5899 895.6877 895.918 895.9247 895.9306
895.9477 896.601 897.6044 898.6131 899.6191 900.6247 901.4714 901.5531 902.5287 902.8335 902.8755 902.9334 903.5259 903.531 904.5689
905.5822 906.4314 906.575 906.5843 907.5222 907.5312 907.5645 907.5691 907.5743 908.4998 908.6497 909.5033 909.6519 910.0163 910.5543
910.6671 910.9028 910.9608 911.6702 912.4555 912.891 912.8995 913.4591 914.4571 915.4561 916.4655 916.4797 916.9108 917.453 917.4944
917.5293 917.5374 917.8914 918.5857 919.5886 920.5405 920.5963 921.4638 921.51 921.5433 921.5992 921.607 921.9446 922.6167 923.5078
923.6237 924.4711 924.6321 925.6352 925.9296 926.5288 926.9387 927.5298 927.9447 928.5724 928.9351 929.0373 929.5706 929.9403 930.0411
930.5544 930.5593 930.9052 931.4483 931.5475 931.555 931.5608 931.8838 931.8889 932.5597 933.5619 934.5156 934.979 935.5174 935.9817
936.5252 936.9122 937.4396 937.5797 937.9163 938.4957 938.9128 938.9198 939.5001 940.4956 940.5056 941.4959 941.5047 942.5254 943.5293
943.9231 944.6025 944.9336 945.5734 946.5221 946.619 947.4992 948.535 949.5388 949.9431 950.4902 950.54 950.953 951.493 951.9549
952.0226 952.4975 952.8882 952.9536 953.1212 953.5019 953.5558 953.8877 954.1245 954.5655 954.8905 955.5703 955.5754 955.893 956.4957

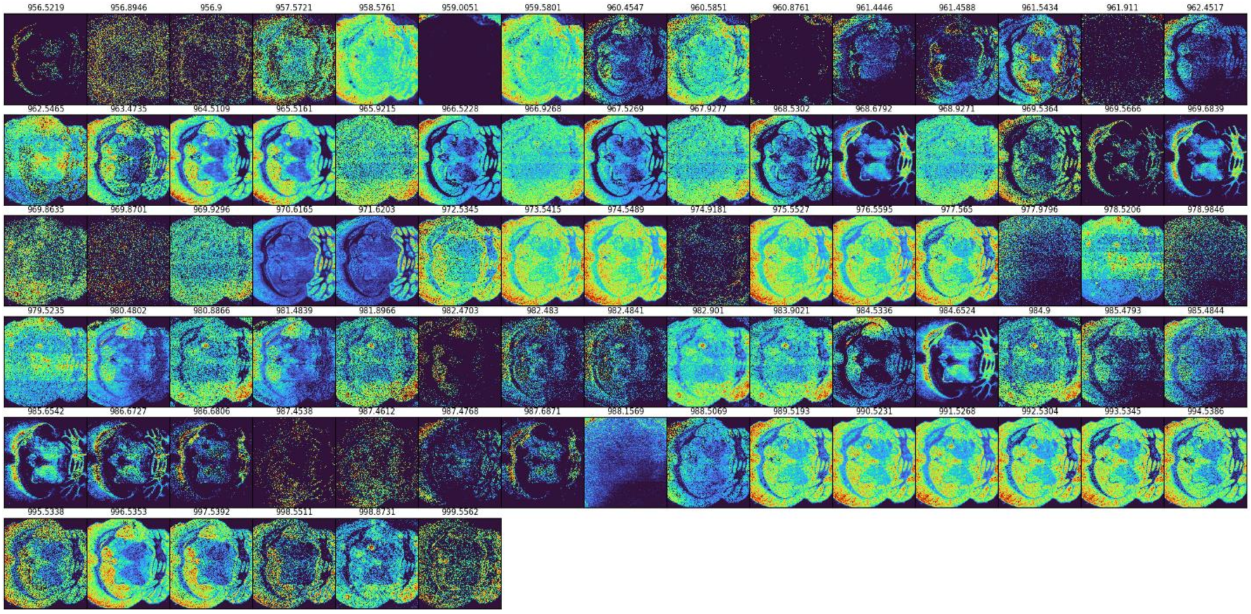

Figure S10. 1101 ion images in the MALDI mouse brain dataset.

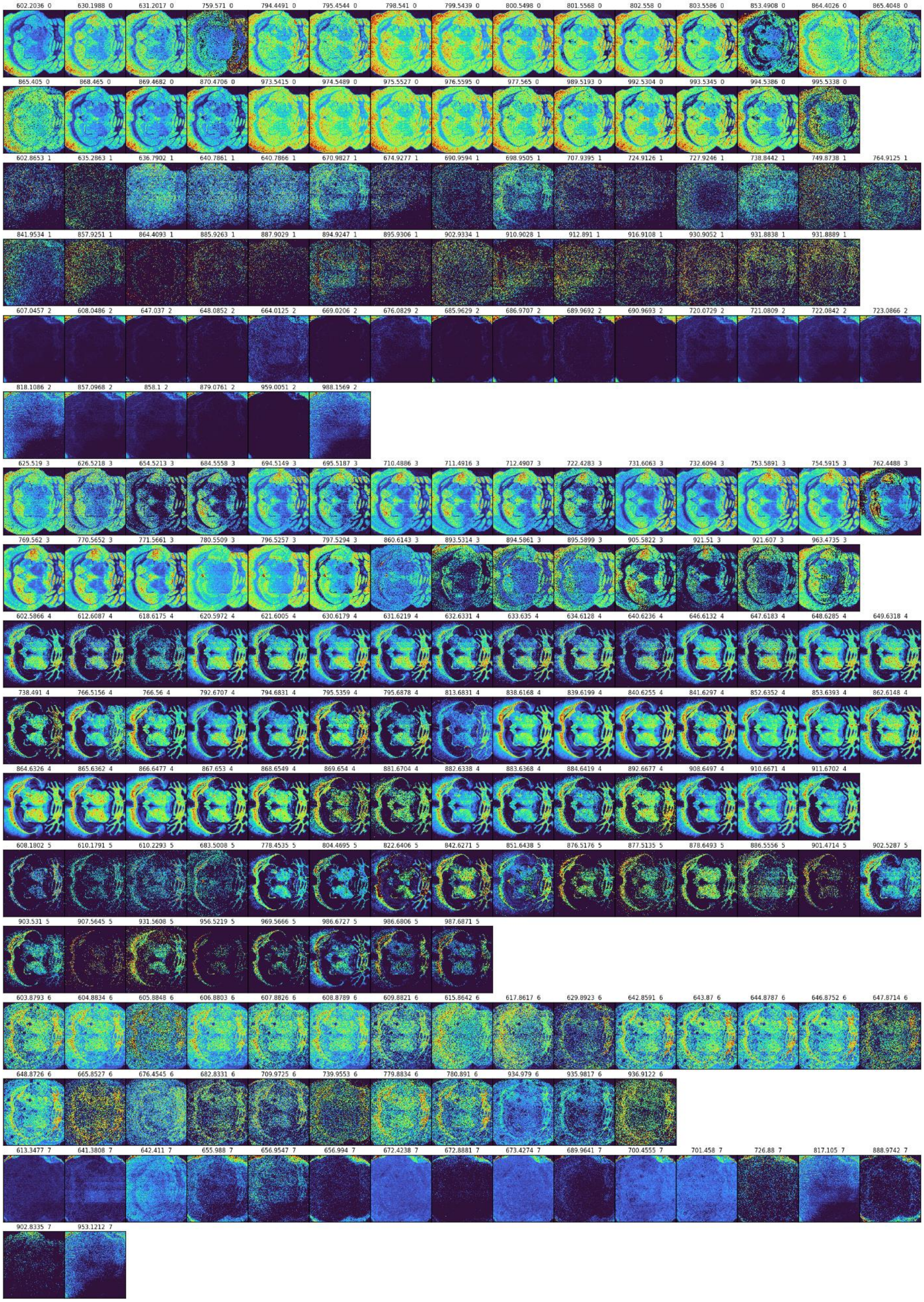
602.2036 0
630.1988 0
631.2017 0
759.571 0
794.4491 0
795.4544 0
798.541 0
799.5439 0
800.5498 0
801.5568 0
802.558 0
803.5586 0
853.4908 0
864.4026 0
865.4048 0
865.405 0
868.465 0
869.4682 0
870.4706 0
973.5415 0
974.5489 0
975.5527 0
976.5595 0
977.565 0
989.5193 0
992.5304 0
993.5345 0
994.5386 0
995.5338 0
602.8653 1
635.2863 1
636.7902 1
640.7861 1
640.7866 1
670.9827 1
674.9277 1
690.9594 1
698.9505 1
707.9395 1
724.9126 1
727.9246 1
738.8442 1
749.8738 1
764.9125 1
841.9534 1
857.9251 1
864.4093 1
885.9263 1
887.9029 1
894.9247 1
895.9306 1
902.9334 1
910.9028 1
912.891 1
916.9108 1
930.9052 1
931.8838 1
931.8889 1
607.0457 2
608.0486 2
647.037 2
648.0852 2
664.0125 2
669.0206 2
676.0829 2
685.9629 2
686.9707 2
689.9692 2
690.9693 2
720.0729 2
721.0809 2
722.0842 2
723.0866 2
818.1086 2
857.0968 2
858.1 2
879.0761 2
959.0051 2
988.1569 2
625.519 3
626.5218 3
654.5213 3
684.5558 3
694.5149 3
695.5187 3
710.4886 3
711.4916 3
712.4907 3
722.4283 3
731.6063 3
732.6094 3
753.5891 3
754.5915 3
762.4488 3
769.562 3
770.5652 3
771.5661 3
780.5509 3
796.5257 3
797.5294 3
860.6143 3
893.5314 3
894.5861 3
895.5899 3
905.5822 3
921.51 3
921.607 3
963.4735 3
602.5866 4
612.6087 4
618.6175 4
620.5972 4
621.6005 4
630.6179 4
631.6219 4
632.6331 4
633.635 4
634.6128 4
640.6236 4
646.6132 4
647.6183 4
648.6285 4
649.6318 4
738.491 4
766.5156 4
766.56 4
792.6707 4
794.6831 4
795.5359 4
795.6878 4
813.6831 4
838.6168 4
839.6199 4
840.6255 4
841.6297 4
852.6352 4
853.6393 4
862.6148 4
864.6326 4
865.6362 4
866.6477 4
867.653 4
868.6549 4
869.654 4
881.6704 4
882.6338 4
883.6368 4
884.6419 4
892.6677 4
908.6497 4
910.6671 4
911.6702 4
608.1802 5
610.1791 5
610.2293 5
683.5008 5
778.4535 5
804.4695 5
822.6406 5
842.6271 5
851.6438 5
876.5176 5
877.5135 5
878.6493 5
886.5556 5
901.4714 5
902.5287 5
903.531 5
907.5645 5
931.5608 5
956.5219 5
969.5666 5
986.6727 5
986.6806 5
987.6871 5
603.8793 6
604.8834 6
605.8848 6
606.8803 6
607.8826 6
608.8789 6
609.8821 6
615.8642 6
617.8617 6
629.8923 6
642.8591 6
643.87 6
644.8787 6
646.8752 6
647.8714 6
648.8726 6
665.8527 6
676.4545 6
682.8331 6
709.9725 6
739.9553 6
779.8834 6
780.891 6
934.979 6
935.9817 6
936.9122 6
613.3477 7
641.3808 7
642.411 7
655.988 7
656.9547 7
656.994 7
672.4238 7
672.8881 7
673.4274 7
689.9641 7
700.4555 7
701.458 7
726.88 7
817.105 7
888.9742 7
902.8335 7
953.1212 7

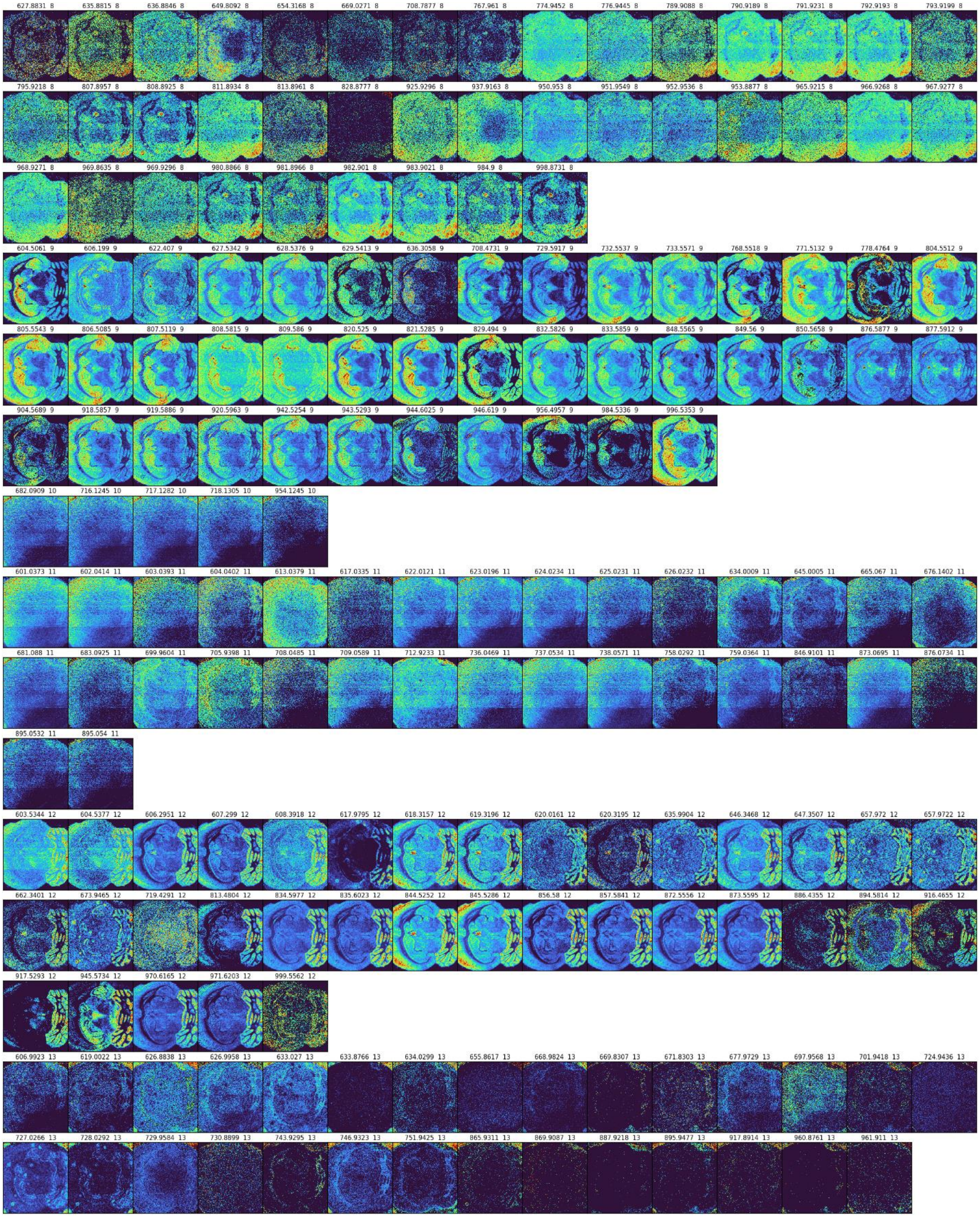
627.8831 8
635.8815 8
636.8846 8
649.8092 8
654.3168 8
669.0271 8
708.7877 8
767.961 8
774.9452 8
776.9445 8
789.9088 8
790.9189 8
791.9231 8
792.9193 8
793.9199 8
795.9218 8
807.8957 8
808.8925 8
811.8934 8
813.8961 8
828.8777 8
925.9296 8
937.9163 8
950.953 8
951.9549 8
952.9536 8
953.8877 8
965.9215 8
966.9268 8
967.9277 8
968.9271 8
969.8635 8
969.9296 8
980.8866 8
981.8966 8
982.901 8
983.9021 8
984.9 8
998.8731 8
604.5061 9
606.199 9
622.407 9
627.5342 9
628.5376 9
629.5413 9
636.3058 9
708.4731 9
729.5917 9
732.5537 9
733.5571 9
768.5518 9
771.5132 9
778.4764 9
804.5512 9
805.5543 9
806.5085 9
807.5119 9
808.5815 9
809.586 9
820.525 9
821.5285 9
829.494 9
832.5826 9
833.5859 9
848.5565 9
849.56 9
850.5658 9
876.5877 9
877.5912 9
904.5689 9
918.5857 9
919.5886 9
920.5963 9
942.5254 9
943.5293 9
944.6025 9
946.619 9
956.4957 9
984.5336 9
996.5353 9
682.0909 10
716.1245 10
717.1282 10
718.1305 10
954.1245 10
601.0373 11
602.0414 11
603.0393 11
604.0402 11
613.0379 11
617.0335 11
622.0121 11
623.0196 11
624.0234 11
625.0231 11
626.0232 11
634.0009 11
645.0005 11
665.067 11
676.1402 11
681.088 11
683.0925 11
699.9604 11
705.9398 11
708.0485 11
709.0589 11
712.9233 11
736.0469 11
737.0534 11
738.0571 11
758.0292 11
759.0364 11
846.9101 11
873.0695 11
876.0734 11
895.0532 11
895.054 11
603.5344 12
604.5377 12
606.2951 12
607.299 12
608.3918 12
617.9795 12
618.3157 12
619.3196 12
620.0161 12
620.3195 12
635.9904 12
646.3468 12
647.3507 12
657.972 12
657.9722 12
662.3401 12
673.9465 12
719.4291 12
813.4804 12
834.5977 12
835.6023 12
844.5252 12
845.5286 12
856.58 12
857.5841 12
872.5556 12
873.5595 12
886.4355 12
894.5814 12
916.4655 12
917.5293 12
945.5734 12
970.6165 12
971.6203 12
999.5562 12
606.9923 13
619.0022 13
626.8838 13
626.9958 13
633.027 13
633.8766 13
634.0299 13
655.8617 13
668.9824 13
669.8307 13
671.8303 13
677.9729 13
697.9568 13
701.9418 13
724.9436 13
727.0266 13
728.0292 13
729.9584 13
730.8899 13
743.9295 13
746.9323 13
751.9425 13
865.9311 13
869.9087 13
887.9218 13
895.9477 13
917.8914 13
960.8761 13
961.911 13

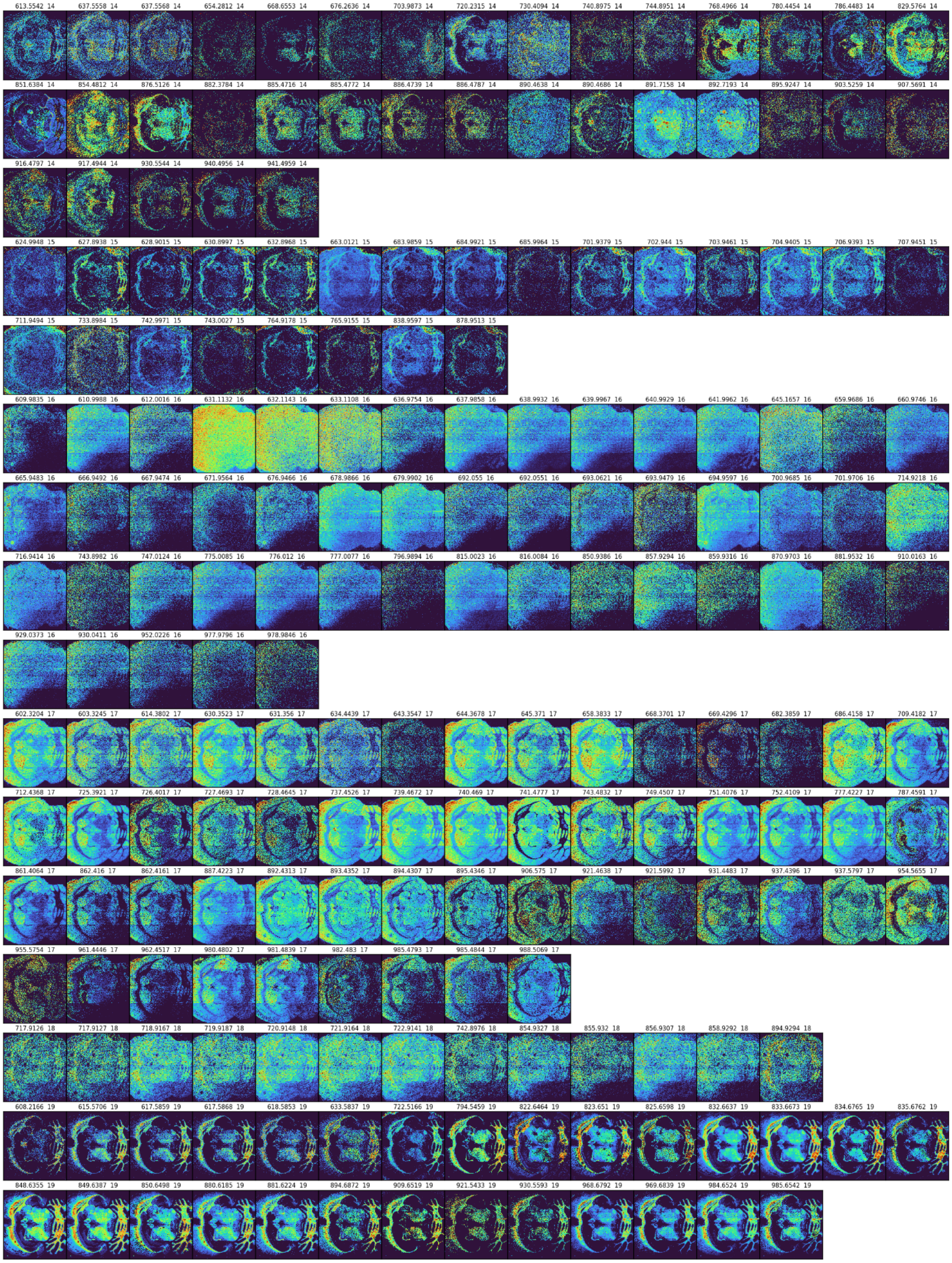
613.5542 14
637.5558 14
637.5568 14
654.2812 14
668.6553 14
676.2636 14
703.9873 14
720.2315 14
730.4094 14
740.8975 14
744.8951 14
768.4966 14
780.4454 14
786.4483 14
829.5764 14
851.6384 14
854.4812 14
876.5126 14
882.3784 14
885.4716 14
885.4772 14
886.4739 14
886.4787 14
890.4638 14
890.4686 14
891.7158 14
892.7193 14
895.9247 14
903.5259 14
907.5691 14
916.4797 14
917.4944 14
930.5544 14
940.4956 14
941.4959 14
624.9948 15
627.8938 15
628.9015 15
630.8997 15
632.8968 15
663.0121 15
683.9859 15
684.9921 15
685.9964 15
701.9379 15
702.944 15
703.9461 15
704.9405 15
706.9393 15
707.9451 15
711.9494 15
733.8984 15
742.9971 15
743.0027 15
764.9178 15
765.9155 15
838.9597 15
878.9513 15
609.9835 16
610.9988 16
612.0016 16
631.1132 16
632.1143 16
633.1108 16
636.9754 16
637.9858 16
638.9932 16
639.9967 16
640.9929 16
641.9962 16
645.1657 16
659.9686 16
660.9746 16
665.9483 16
666.9492 16
667.9474 16
671.9564 16
676.9466 16
678.9866 16
679.9902 16
692.055 16
692.0551 16
693.0621 16
693.9479 16
694.9597 16
700.9685 16
701.9706 16
714.9218 16
716.9414 16
743.8982 16
747.0124 16
775.0085 16
776.012 16
777.0077 16
796.9894 16
815.0023 16
816.0084 16
850.9386 16
857.9294 16
859.9316 16
870.9703 16
881.9532 16
910.0163 16
929.0373 16
930.0411 16
952.0226 16
977.9796 16
978.9846 16
602.3204 17
603.3245 17
614.3802 17
630.3523 17
631.356 17
634.4439 17
643.3547 17
644.3678 17
645.371 17
658.3833 17
668.3701 17
669.4296 17
682.3859 17
686.4158 17
709.4182 17
712.4368 17
725.3921 17
726.4017 17
727.4693 17
728.4645 17
737.4526 17
739.4672 17
740.469 17
741.4777 17
743.4832 17
749.4507 17
751.4076 17
752.4109 17
777.4227 17
787.4591 17
861.4064 17
862.416 17
862.4161 17
887.4223 17
892.4313 17
893.4352 17
894.4307 17
895.4346 17
906.575 17
921.4638 17
921.5992 17
931.4483 17
937.4396 17
937.5797 17
954.5655 17
955.5754 17
961.4446 17
962.4517 17
980.4802 17
981.4839 17
982.483 17
985.4793 17
985.4844 17
988.5069 17
717.9126 18
717.9127 18
718.9167 18
719.9187 18
720.9148 18
721.9164 18
722.9141 18
742.8976 18
854.9327 18
855.932 18
856.9307 18
858.9292 18
894.9294 18
608.2166 19
615.5706 19
617.5859 19
617.5868 19
618.5853 19
633.5837 19
722.5166 19
794.5459 19
822.6464 19
823.651 19
825.6598 19
832.6637 19
833.6673 19
834.6765 19
835.6762 19
848.6355 19
849.6387 19
850.6498 19
880.6185 19
881.6224 19
894.6872 19
909.6519 19
921.5433 19
930.5593 19
968.6792 19
969.6839 19
984.6524 19
985.6542 19

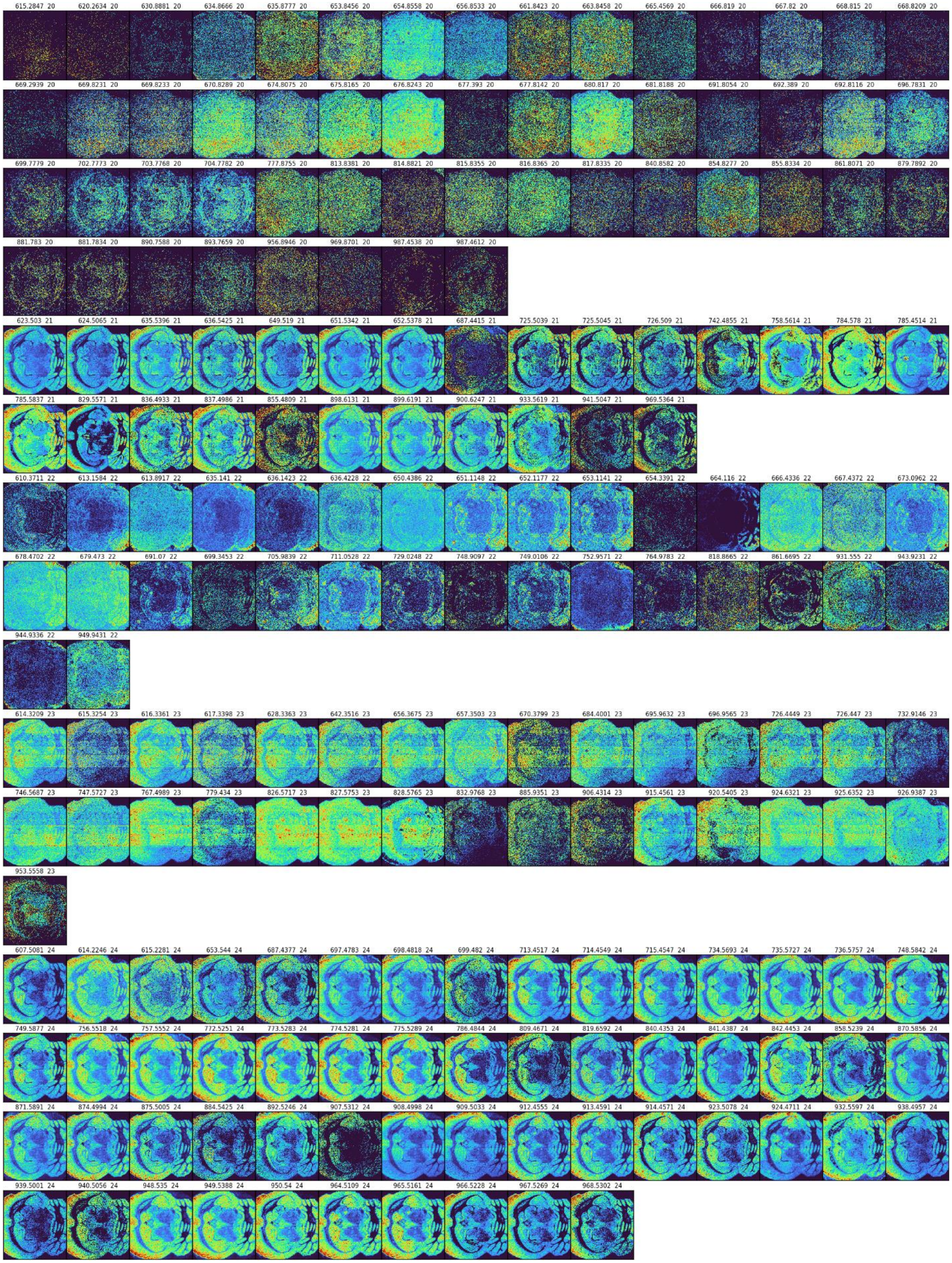
615.2847 20
620.2634 20
630.8881 20
634.8666 20
635.8777 20
653.8456 20
654.8558 20
656.8533 20
661.8423 20
663.8458 20
665.4569 20
666.819 20
667.82 20
668.815 20
668.8209 20
669.2939 20
669.8231 20
669.8233 20
670.8289 20
674.8075 20
675.8165 20
676.8243 20
677.393 20
677.8142 20
680.817 20
681.8188 20
691.8054 20
692.389 20
692.8116 20
696.7831 20
699.7779 20
702.7773 20
703.7768 20
704.7782 20
777.8755 20
813.8381 20
814.8821 20
815.8355 20
816.8365 20
817.8335 20
840.8582 20
854.8277 20
855.8334 20
861.8071 20
879.7892 20
881.783 20
881.7834 20
890.7588 20
893.7659 20
956.8946 20
969.8701 20
987.4538 20
987.4612 20
623.503 21
624.5065 21
635.5396 21
636.5425 21
649.519 21
651.5342 21
652.5378 21
687.4415 21
725.5039 21
725.5045 21
726.509 21
742.4855 21
758.5614 21
784.578 21
785.4514 21
785.5837 21
829.5571 21
836.4933 21
837.4986 21
855.4809 21
898.6131 21
899.6191 21
900.6247 21
933.5619 21
941.5047 21
969.5364 21
610.3711 22
613.1584 22
613.8917 22
635.141 22
636.1423 22
636.4228 22
650.4386 22
651.1148 22
652.1177 22
653.1141 22
654.3391 22
664.116 22
666.4336 22
667.4372 22
673.0962 22
678.4702 22
679.473 22
691.07 22
699.3453 22
705.9839 22
711.0528 22
729.0248 22
748.9097 22
749.0106 22
752.9571 22
764.9783 22
818.8665 22
861.6695 22
931.555 22
943.9231 22
944.9336 22
949.9431 22
614.3209 23
615.3254 23
616.3361 23
617.3398 23
628.3363 23
642.3516 23
656.3675 23
657.3503 23
670.3799 23
684.4001 23
695.9632 23
696.9565 23
726.4449 23
726.447 23
732.9146 23
746.5687 23
747.5727 23
767.4989 23
779.434 23
826.5717 23
827.5753 23
828.5765 23
832.9768 23
885.9351 23
906.4314 23
915.4561 23
920.5405 23
924.6321 23
925.6352 23
926.9387 23
953.5558 23
607.5081 24
614.2246 24
615.2281 24
653.544 24
687.4377 24
697.4783 24
698.4818 24
699.482 24
713.4517 24
714.4549 24
715.4547 24
734.5693 24
735.5727 24
736.5757 24
748.5842 24
749.5877 24
756.5518 24
757.5552 24
772.5251 24
773.5283 24
774.5281 24
775.5289 24
786.4844 24
809.4671 24
819.6592 24
840.4353 24
841.4387 24
842.4453 24
858.5239 24
870.5856 24
871.5891 24
874.4994 24
875.5005 24
884.5425 24
892.5246 24
907.5312 24
908.4998 24
909.5033 24
912.4555 24
913.4591 24
914.4571 24
923.5078 24
924.4711 24
932.5597 24
938.4957 24
939.5001 24
940.5056 24
948.535 24
949.5388 24
950.54 24
964.5109 24
965.5161 24
966.5228 24
967.5269 24
968.5302 24

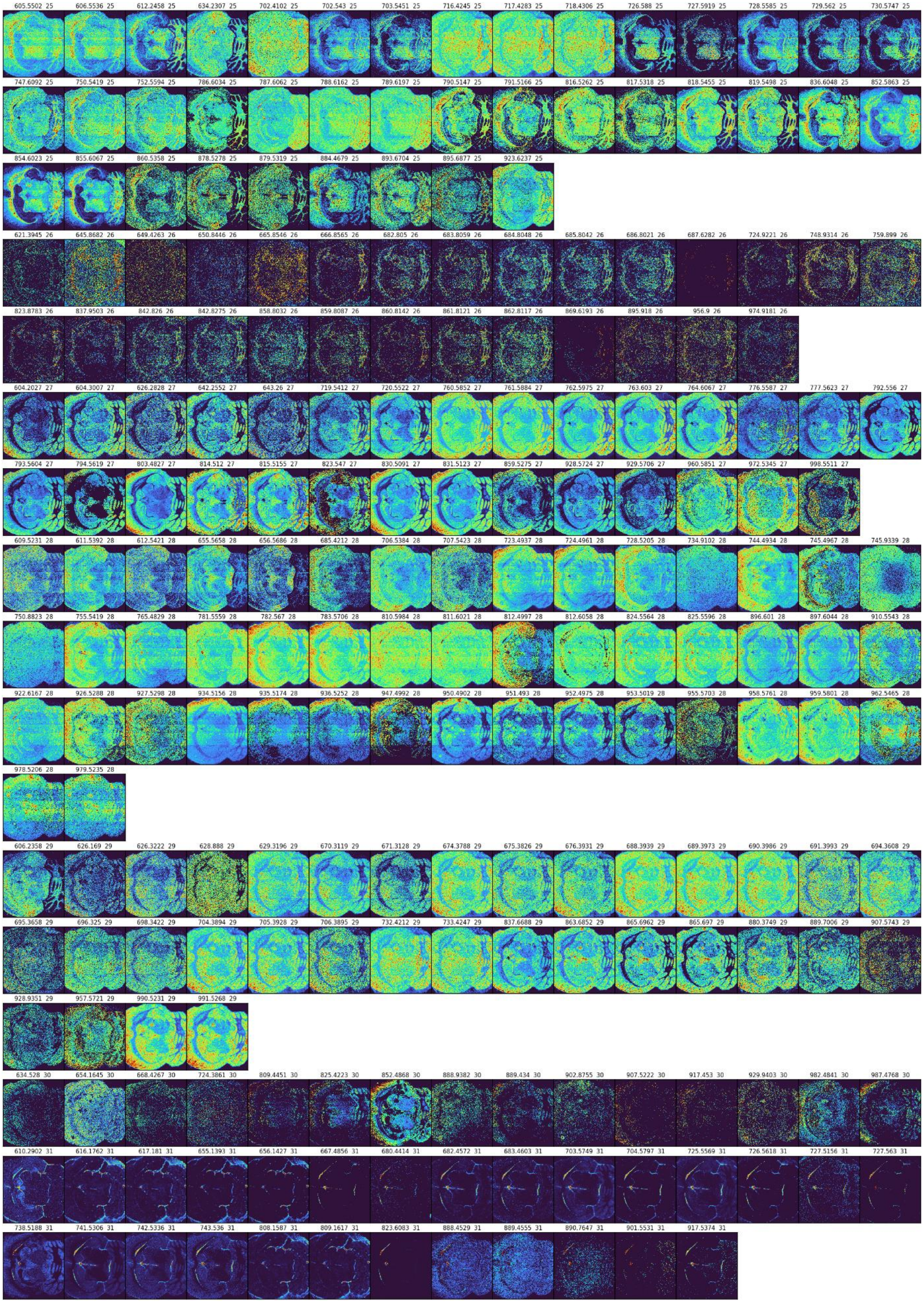
605.5502 25
606.5536 25
612.2458 25
634.2307 25
702.4102 25
702.543 25
703.5451 25
716.4245 25
717.4283 25
718.4306 25
726.588 25
727.5919 25
728.5585 25
729.562 25
730.5747 25
747.6092 25
750.5419 25
752.5594 25
786.6034 25
787.6062 25
788.6162 25
789.6197 25
790.5147 25
791.5166 25
816.5262 25
817.5318 25
818.5455 25
819.5498 25
836.6048 25
852.5863 25
854.6023 25
855.6067 25
860.5358 25
878.5278 25
879.5319 25
884.4679 25
893.6704 25
895.6877 25
923.6237 25
621.3945 26
645.8682 26
649.4263 26
650.8446 26
665.8546 26
666.8565 26
682.805 26
683.8059 26
684.8048 26
685.8042 26
686.8021 26
687.6282 26
724.9221 26
748.9314 26
759.899 26
823.8783 26
837.9503 26
842.826 26
842.8275 26
858.8032 26
859.8087 26
860.8142 26
861.8121 26
862.8117 26
869.6193 26
895.918 26
956.9 26
974.9181 26
604.2027 27
604.3007 27
626.2828 27
642.2552 27
643.26 27
719.5412 27
720.5522 27
760.5852 27
761.5884 27
762.5975 27
763.603 27
764.6067 27
776.5587 27
777.5623 27
792.556 27
793.5604 27
794.5619 27
803.4827 27
814.512 27
815.5155 27
823.547 27
830.5091 27
831.5123 27
859.5275 27
928.5724 27
929.5706 27
960.5851 27
972.5345 27
998.5511 27
609.5231 28
611.5392 28
612.5421 28
655.5658 28
656.5686 28
685.4212 28
706.5384 28
707.5423 28
723.4937 28
724.4961 28
728.5205 28
734.9102 28
744.4934 28
745.4967 28
745.9339 28
750.8823 28
755.5419 28
765.4829 28
781.5559 28
782.567 28
783.5706 28
810.5984 28
811.6021 28
812.4997 28
812.6058 28
824.5564 28
825.5596 28
896.601 28
897.6044 28
910.5543 28
922.6167 28
926.5288 28
927.5298 28
934.5156 28
935.5174 28
936.5252 28
947.4992 28
950.4902 28
951.493 28
952.4975 28
953.5019 28
955.5703 28
958.5761 28
959.5801 28
962.5465 28
978.5206 28
979.5235 28
606.2358 29
626.169 29
626.3222 29
628.888 29
629.3196 29
670.3119 29
671.3128 29
674.3788 29
675.3826 29
676.3931 29
688.3939 29
689.3973 29
690.3986 29
691.3993 29
694.3608 29
695.3658 29
696.325 29
698.3422 29
704.3894 29
705.3928 29
706.3895 29
732.4212 29
733.4247 29
837.6688 29
863.6852 29
865.6962 29
865.697 29
880.3749 29
889.7006 29
907.5743 29
928.9351 29
957.5721 29
990.5231 29
991.5268 29
634.528 30
654.1645 30
668.4267 30
724.3861 30
809.4451 30
825.4223 30
852.4868 30
888.9382 30
889.434 30
902.8755 30
907.5222 30
917.453 30
929.9403 30
982.4841 30
987.4768 30
610.2902 31
616.1762 31
617.181 31
655.1393 31
656.1427 31
667.4856 31
680.4414 31
682.4572 31
683.4603 31
703.5749 31
704.5797 31
725.5569 31
726.5618 31
727.5156 31
727.563 31
738.5188 31
741.5306 31
742.5336 31
743.536 31
808.1587 31
809.1617 31
823.6083 31
888.4529 31
889.4555 31
890.7647 31
901.5531 31
917.5374 31

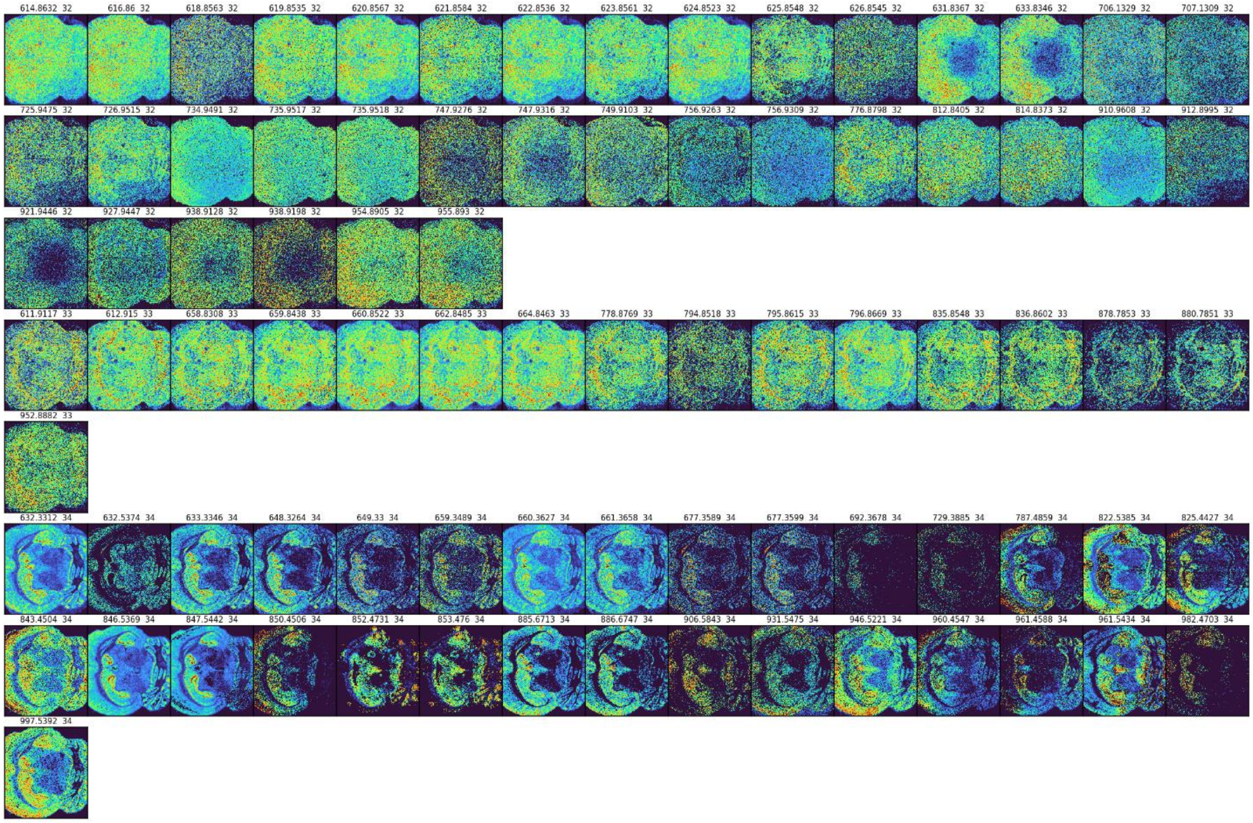

Figure S11. Self-supervised clustering results of 1101 ion images in the MALDI mouse brain dataset.

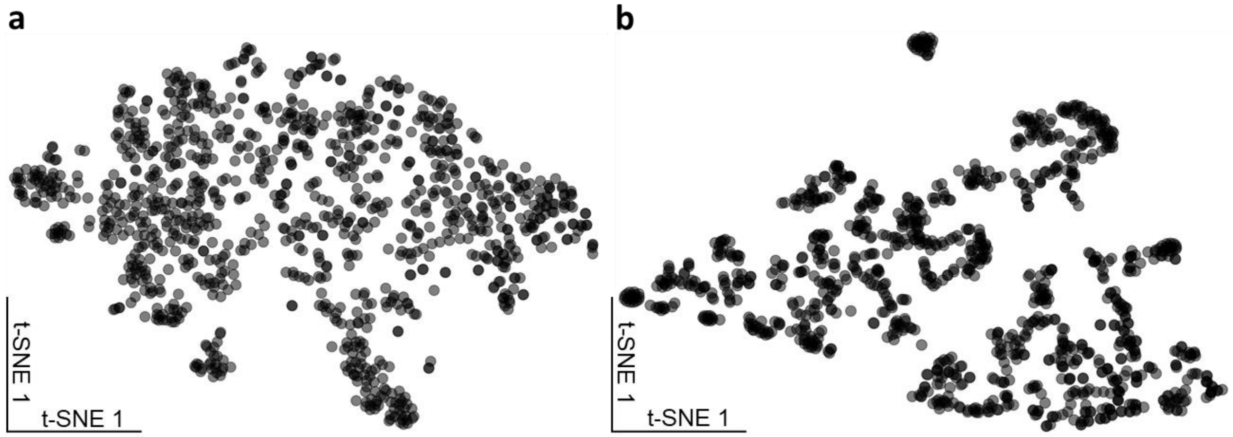

Figure S12. t-SNE visualizations of ion image representations obtained from (a) EfficientNet-B0 and (b) re-trained CNN encoder.

References

1. Yin, R., Burnum-johnson, K. E. & Laskin, J. High spatial resolution imaging of biological tissues using nanospray desorption electrospray ionization mass spectrometry. *Nat. Protoc.* 14, 3445–3470. (2019).

2. Kompauer, M., Heiles, S. & Spengler, B. Atmospheric pressure MALDI mass spectrometry imaging of tissues and cells at 1.4-µm lateral resolution. *Nat. Methods* 14, 90–96 (2016).

3. Yin, R. *et al.* High Spatial Resolution Imaging of Mouse Pancreatic Islets Using Nanospray Desorption Electrospray Ionization Mass Spectrometry. *Anal. Chem.* 90, 6548–6555 (2018).

4. Palmer, A. *et al.* FDR-controlled metabolite annotation for high-resolution imaging mass spectrometry. *Nat. Methods* 14, 57–60 (2016).

5. Hu, H., Yin, R., Brown, H. M. & Laskin, J. Spatial Segmentation of Mass Spectrometry Imaging Data by Combining Multivariate Clustering and Univariate Thresholding. *Anal. Chem.* 93, 3477–3485 (2021).

6. Chen, T., Kornblith, S., Norouzi, M. & Hinton, G. A simple framework for contrastive learning of visual representations. In *Int. Con. on Mach. Learn.* 1575–1585 (ICML 2020).

7. Van Gansbeke, W., Vandenhende, S., Georgoulis, S., Proesmans, M. & Van Gool, L. SCAN: Learning to Classify Images Without Labels. In *Eur. Conf. on Comput. Vis.* 268–285 (ECCV 2020).

8. Von Luxburg, U. A tutorial on spectral clustering. *Stat. Comput.* 17, 395–416 (2007).

9. Smets, T.; Waelkens, E.; De Moor, B. Prioritization of m / z -Values in Mass Spectrometry Imaging Profiles Obtained Using Uniform Manifold Approximation and Projection for Dimensionality Reduction . *Anal. Chem.* **2020**, *92* (7), 5240–5248.

10. Zhang, W. *et al.* Spatially aware clustering of ion images in mass spectrometry imaging data using deep learning. *Anal. Bioanal. Chem.* 413, 2803–2819 (2021).
